# Glypican Is a Modulator of Netrin-Mediated Axon Guidance

**DOI:** 10.1371/journal.pbio.1002183

**Published:** 2015-07-06

**Authors:** Cassandra R. Blanchette, Paola N. Perrat, Andrea Thackeray, Claire Y. Bénard

**Affiliations:** Department of Neurobiology, University of Massachusetts Medical School, Worcester, Massachusetts, United States of America; Johns Hopkins University School of Medicine, UNITED STATES

## Abstract

Netrin is a key axon guidance cue that orients axon growth during neural circuit formation. However, the mechanisms regulating netrin and its receptors in the extracellular milieu are largely unknown. Here we demonstrate that in *Caenorhabditis elegans*, LON-2/glypican, a heparan sulfate proteoglycan, modulates UNC-6/netrin signaling and may do this through interactions with the UNC-40/DCC receptor. We show that developing axons misorient in the absence of LON-2/glypican when the SLT-1/slit guidance pathway is compromised and that LON-2/glypican functions in both the attractive and repulsive UNC-6/netrin pathways. We find that the core LON-2/glypican protein, lacking its heparan sulfate chains, and secreted forms of LON-2/glypican are functional in axon guidance. We also find that LON-2/glypican functions from the epidermal substrate cells to guide axons, and we provide evidence that LON-2/glypican associates with UNC-40/DCC receptor–expressing cells. We propose that LON-2/glypican acts as a modulator of UNC-40/DCC-mediated guidance to fine-tune axonal responses to UNC-6/netrin signals during migration.

## Abstract 2

Work in nematodes shows that the proteoglycan glypican acts as an extracellular modulator of netrin signals during nervous system development, interacting with cells that express the netrin receptor.

## Author Summary

During the development of the nervous system, migrating axons are guided as they navigate through complex environments to reach their target destinations. These directed migrations are essential to ensure the proper wiring and function of the nervous system and are instructed by guidance cues and receptors. There is a remarkably small set of guidance cues and receptors relative to the large number of neuronal migrations, suggesting that the actions of these guidance cues might be diversified by regulatory mechanisms. We have addressed this question in the genetically tractable nematode *Caenorhabditis elegans*. We identify that the response of migrating neurons to a key guidance cue, UNC-6/netrin, is modulated by a specific proteoglycan, LON-2/glypican. We show that LON-2/glypican may carry out this regulation by interacting with the UNC-40/DCC netrin receptor on the cell surface. We propose that LON-2/glypican acts as an extracellular modulator of UNC-40/DCC-mediated guidance to fine-tune axonal responses to UNC-6/netrin signals during migration.

## Introduction

Directed migrations of developing axons are essential for the proper wiring of the nervous system. A host of guidance cues and their receptors instruct axon guidance decisions. However, how these cues and the growth cone’s responses to them are spatially and temporally regulated in vivo remains largely unknown. Answering this question is central to our understanding of how growing axons navigate in complex environments to reach their targets during development and regeneration.

UNC-6/netrin is a highly conserved secreted guidance cue with structural similarity to the extracellular matrix protein laminin [1–3]. UNC-6/netrin directs attractive guidance through receptors of the UNC-40/DCC family and repulsive guidance through both UNC-40/DCC and UNC-5/UNC5 receptors [4–6]. Notably, whereas netrin receptors and downstream transduction pathways have been well characterized, how netrin signals are regulated extracellularly remains largely unknown. UNC-6/netrin was identified through genetic analysis in *Caenorhabditis elegans* [1] and biochemically purified and cloned from vertebrate embryos [2]. A second biochemical component that synergized with netrin to elicit axon outgrowth was termed “netrin synergizing activity” (NSA) [3] and remains unidentified. Vertebrate netrin-1 and its receptor DCC can bind heparin, a fully sulfated version of heparan sulfate (HS), in vitro [3,7,8], and a general disruption of HS chain synthesis is detrimental to netrin-1-mediated axon outgrowth in vitro [9,10]. While heparan sulfate proteoglycans (HSPGs) might be intriguing candidates for NSA, it is not yet known whether a specific HSPG is required for netrin signaling or how interactions with HSPGs might regulate netrin signals to direct axons during nervous system development.

**Fig 1:**
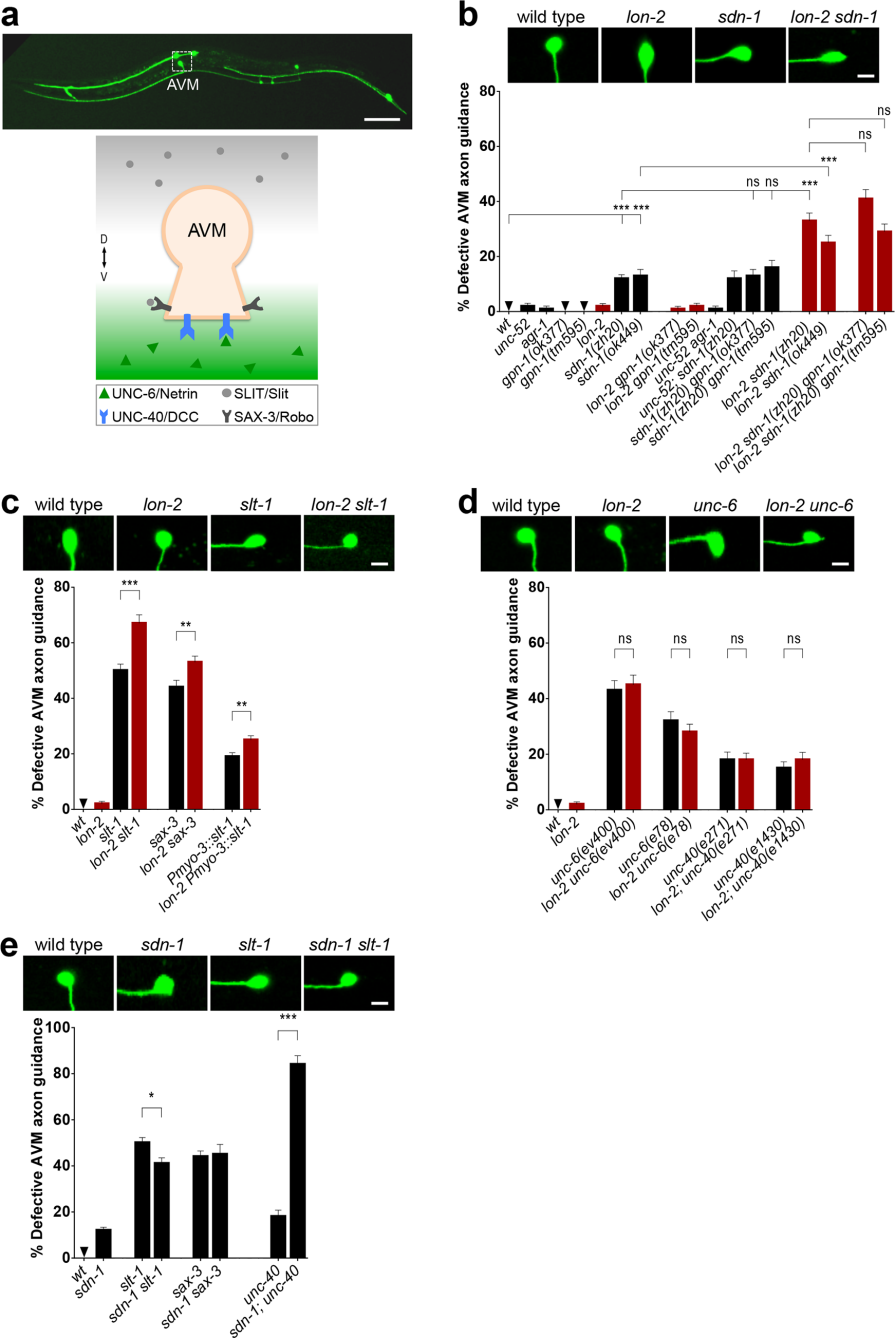
*lon-2*/glypican functions in the attractive *unc-6*/netrin guidance pathway. (A) During the first larval stage of *C*. *elegans*, the pioneer neuron AVM extends ventrally along the body wall until it reaches the ventral nerve cord. Its migration results from the combined attractive response to UNC-6/netrin (secreted at the ventral midline) via the UNC-40/DCC receptor and the repulsive response to SLT-1/Slit (secreted by the dorsal muscles) via its SAX-3/Robo receptor. We visualized the morphology of the AVM axon using the transgene P*mec-4*::*gfp*. (B) The heparan sulfate proteoglycans *lon-2*/glypican and *sdn-1*/syndecan cooperate to guide the axon of AVM, as their simultaneous loss enhances guidance defects. The role of *lon-2*/glypican in axon guidance is specific, as the loss of *lon-2*/glypican, but not the loss of the other *C*. *elegans* glypican, *gpn-1*, enhances the defects of *sdn-1*/syndecan mutants. (C) Complete loss of *lon-2*/glypican enhances the axon guidance defects resulting from disrupted *slt-1*/Slit signaling in mutants for *slt-1*/Slit or its receptor *sax-3*/Robo, as well as in animals misexpressing *slt-1* in all body wall muscles (using a P*myo-3*::*slt-1* transgene). Data for wild type and *lon-2* are the same as in (B). (D) Complete loss of *lon-2*/glypican does not enhance the AVM guidance defects of *unc-6*/netrin mutants or of mutants for its receptor *unc-40*/DCC, suggesting that *lon-2*/glypican functions in the same genetic pathway as *unc-6*/netrin. Data for wild type and *lon-2* are the same as in (B). (E) Loss of *sdn-1*/syndecan function does not enhance the defects of *slt-1*/Slit or sax-3/Robo mutants but enhances the defects of *unc-*40/DCC mutants. Data for wild type, *sdn-1*, *slt-1*, *sax-3*, and *unc-40* are the same as in (B–D). Error bars are standard error of the proportion. Asterisks denote significant difference\: *** *p* ≤ 0.001,** *p* ≤ 0.01, and * *p* ≤ 0.05 (*z*-tests, *p*-values were corrected by multiplying by the number of comparisons). ns, not significant.

We addressed these questions using the nematode *C*. *elegans*, which has been instrumental for discovering major conserved axon guidance pathways. During larval development, the axon of the mechanosensory neuron AVM migrates ventrally as its growth cone integrates signals from two complementary guidance cues (Fig 1A) [1,4–6, 11–13]: (1) UNC-6/netrin is secreted at the ventral midline and attracts the growth cone ventrally via the receptor UNC-40/DCC [5,14], and (2) SLT-1/Slit is secreted by the dorsal muscles and repels the growth cone away from the dorsal side via the receptor SAX-3/Robo [12,13]. Animals null for the guidance cues *unc-6*/netrin or *slt-1*/Slit exhibit partial AVM ventral axon guidance defects, and loss of both cues in *unc-6 slt-1* double mutants results in fully penetrant guidance defects (S1 Fig, [13]). AVM axons defective in guidance fail to extend ventrally and instead migrate laterally in the anterior direction (Fig 1). In this study, we use the AVM axon as a model to elucidate mechanisms that regulate UNC-6/netrin signaling.

Here we provide a missing link in understanding the modulation of UNC-6/netrin signaling in the extracellular milieu. We demonstrate that LON-2/glypican, a HSPG secreted from epidermal cells, acts as a modulator of the UNC-6/netrin signaling pathways to guide migrating cells and axons. We show that LON-2/glypican modulates UNC-6/netrin signaling in both attractive guidance mediated by the UNC-40/DCC receptor and repulsive guidance mediated by the UNC-40/DCC and UNC-5/UNC5 receptors. We provide evidence that LON-2/glypican associates with UNC-40/DCC-receptor-expressing cells. We show that the N-terminal globular region of LON-2/glypican, lacking the three HS chain attachment sites, is functional in UNC-6/netrin-mediated guidance. Our studies unravel a novel mechanism by which LON-2/glypican is produced by substrate epidermal cells and released from the membrane to likely associate with UNC-40/DCC-expressing neurons, enabling the modulation of their responses to UNC-6/netrin during axon migrations.

## Results

### *lon-2*/Glypican and *sdn-1*/Syndecan Cooperate to Guide *unc-6*/Netrin- and *slt-1*/Slit-Mediated Axon Migration

To address whether a specific HSPG interacts with the netrin signaling system to guide axons, we first examined axon guidance in mutants lacking core HSPGs. HSPGs are composed of a core protein with covalently attached long unbranched HS chains [15]. HSPGs can be associated with the plasma membrane through either a transmembrane domain (e.g., syndecans) or a glycerophosphatidylinositide (GPI) anchor (e.g., glypicans) or be secreted into the extracellular milieu (e.g., perlecans and agrins). We examined the axon morphology of AVM in single, double, and triple mutants for several core HSPG proteins (see S1 Table for alleles). These included the sole *C*. *elegans* syndecan (*sdn-1*), the two glypicans (*lon-2* and *gpn-1)*, perlecan (*unc-52)*, and agrin (*agr-1*). We found that the mild AVM axon guidance defects of *sdn-1*/syndecan mutants, including a null, [16] were enhanced by the complete loss of *lon-*2/glypican in double mutants *lon-2 sdn-1* (Fig 1B), revealing a role for *lon-2*/glypican in AVM axon guidance. Similarly, loss of *lon-2/*glypican enhances *sdn-1/*syndecan mutants in motorneuron guidance [17]. Although the *C*. *elegans* genome encodes two glypicans, loss of function of the second glypican, *gpn-1*, using two likely null mutant alleles (see S2 Fig), did not enhance the defects of *lon-2*/glypican or *sdn-1*/syndecan null mutants in double or triple mutants (Fig 1B). Moreover, we did not observe abnormal phenotypes in the single mutants for *agr-1*/agrin or *unc-52/*perlecan. These observations highlight the specificity of *lon-2*/glypican function in this axon guidance process and raise the possibility that *lon-2*/glypican might be a component of the pathways guiding the AVM axon towards the ventral midline.

### *lon-2*/Glypican Acts in the Attractive and Repulsive *unc-6*/Netrin Guidance Pathways

Considering that AVM axon guidance occurs via the *unc-6/*netrin and *slt-1/*Slit pathways, mutations in genes such as *lon-2/*glypican and *sdn-1/*syndecan that affect AVM axon guidance may point towards interactions with either of these two guidance systems. Since the AVM axon guidance defects in *lon-2 sdn-1* double mutants are qualitatively similar to those of mutants lacking *unc-6*/netrin or *slt-1*/Slit, we determined how *lon-2*/glypican and *sdn-1*/syndecan impact *unc-6*/netrin and *slt-1*/Slit signaling. In animals that completely lack *slt-*1/Slit function, the complete loss of a gene functioning independently of *slt-*1/Slit is expected to enhance the AVM guidance defects, such as in the double null mutants *unc-6/*netrin *slt-1*/Slit (see S1 Fig). We tested the interactions of *lon-2*/glypican with the *slt-1*/Slit pathway in AVM axon guidance and found that the complete loss of *lon-2*/glypican enhanced a presumptive null allele of *slt-1*/Slit in *lon-2 slt-1* double mutants (Fig 1C), suggesting that *lon-2*/glypican functions in a pathway separate from *slt-1*/Slit. Loss of *lon-2*/glypican also enhanced guidance defects when signaling through *sax-3*/Robo, the *slt-1*/Slit receptor, was disrupted in *lon-2 sax-3* double null mutants, providing further evidence that *lon-2*/glypican functions in a pathway separate from that of *slt-1*/Slit (Fig 1C). As an additional method to investigate the impact of lacking *lon-2*/glypican function when *slt-1*/Slit signaling is perturbed, we used a transgene that ectopically expresses *slt-1*/Slit from both ventral and dorsal body wall muscles (using P*myo-3*::*slt-1*) and misguides the axon of AVM [18]. Loss of *lon-2*/glypican enhanced the defects caused by *slt-1*/Slit misexpression (Fig 1C), consistent with the above findings that *lon-2*/glypican mediates its axon guidance effects independently of *slt-1*/Slit.

The *unc-6*/netrin pathway functions independently of *slt-1*/Slit to guide AVM. To address whether *lon-2*/glypican functions in the *unc-6*/netrin axon guidance pathway, we examined the AVM axon in double mutants of *lon-2*/glypican and *unc-6*/netrin. In animals that completely lack *unc-6/*netrin function, the complete loss of a gene functioning in the same *unc-6/*netrin pathway is expected to not enhance the AVM guidance defects, such as in the double null mutants *unc-6*; *unc-40* (see S1 Fig). We found that the complete loss of *lon-2*/glypican did not enhance the guidance defects displayed by *unc-6*/netrin null mutants *ev400* (Fig 1D). Given that loss of *lon-2* enhances the defects of other guidance mutants (see doubles with *sdn-1*, *slt-1*, *sax-3*, and P*myo-3*::*slt-1* in Fig 1B and 1C and *sqv-5* in S3 Fig), the lack of enhancement when combined with the *unc-6* null mutation suggests that *lon-2*/glypican functions in the same pathway as *unc-6*/netrin. Consistent with this idea, we also found that complete loss of *lon-2*/glypican did not enhance the AVM guidance defects of two null mutant alleles of the netrin receptor *unc-40*/DCC in the double mutants *unc-40; lon-2* (Fig 1D), suggesting that *lon-2*/glypican functions in the same pathway as *unc-40*/DCC in AVM ventral guidance. These observations raise the interesting possibility that *lon-2*/glypican may be the HSPG dedicated to modulate *unc-6*/netrin signaling through *unc-40/*DCC during axon guidance.

Since *lon-2*/glypican functions independently of *slt-1*/Slit (Fig 1C) and partly separate from *sdn-1*/syndecan (Fig 1B), we tested whether *sdn-1*/syndecan and *slt-1*/Slit function together to guide the axon of AVM. We found that defects in *slt-1 sdn-1* double null mutants were not enhanced compared to the single mutants (Fig 1E), consistent with findings in *Drosophila* [19,20] and *C*. *elegans* [16]. We also found that double null mutants for *sdn-1*/syndecan and the s*lt-1*/Slit receptor *sax-3*/Robo were not enhanced compared to the single mutants (Fig 1E). Our results support the notion that *sdn-1*/syndecan acts in the same genetic pathway as *slt-1*/Slit to guide AVM. Consistent with this, we found that the double null mutants for *sdn-1*/syndecan and the netrin receptor *unc-40*/DCC were enhanced, indicating that *sdn-1*/syndecan functions in a pathway separate from *unc-6*/netrin. The analysis of axon guidance in double mutants of *unc-6*/netrin and *sdn-1*/syndecan was precluded by their lethality. Our results are consistent with the notion that *unc-6*/netrin and *sdn-1*/syndecan act in different pathways of axon guidance.

**Fig 2:**
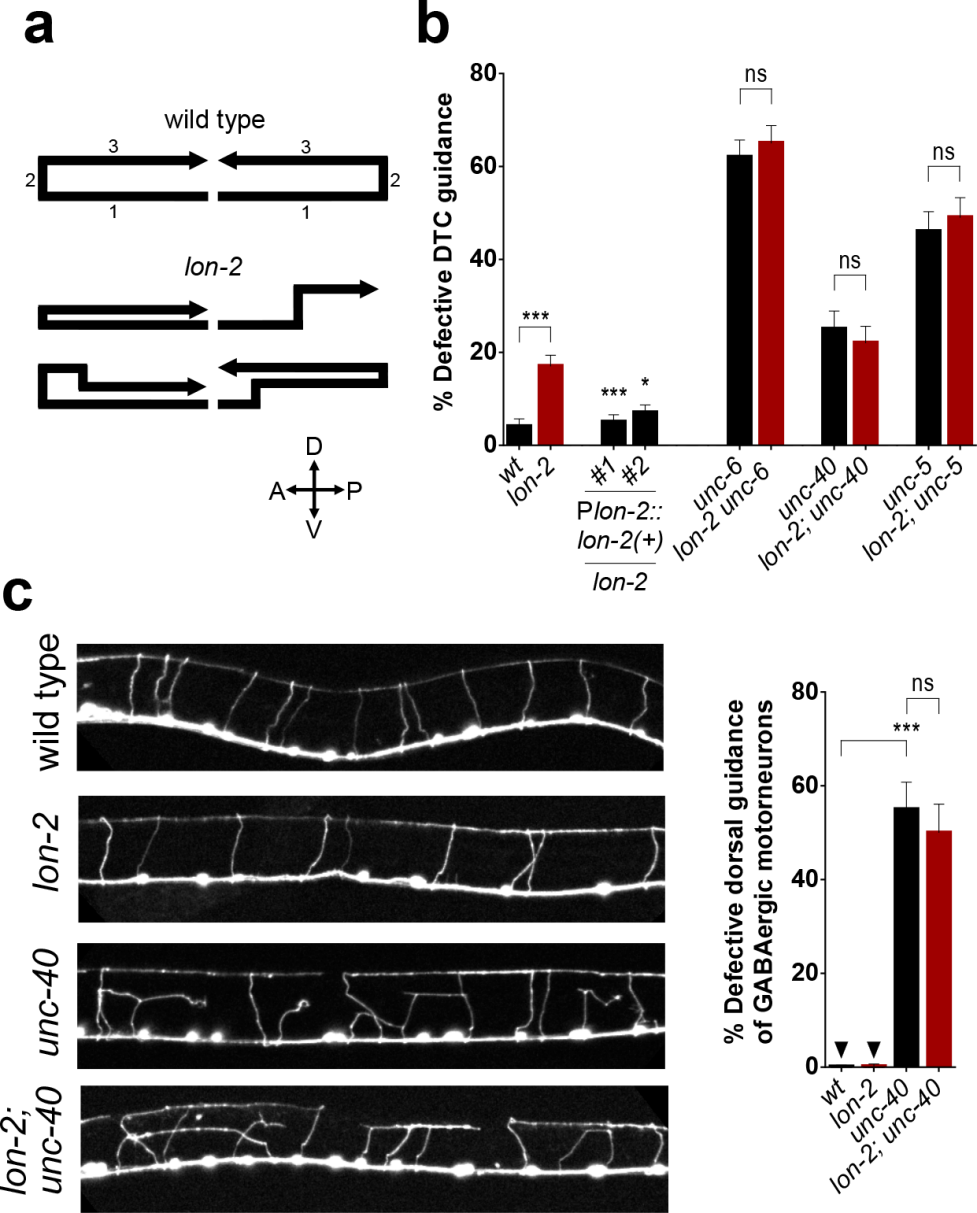
*lon-2/*glypican functions in the repulsive *unc-6*/netrin guidance pathway. (A) Schematics of the migration path of the DTCs in the wild type and examples of defective DTC migration in *lon-2*/glypican mutants (the anterior and the posterior DTCs exhibit similar defects). In wild-type animals, the DTCs migrate away from the vulva along the anteroposterior axis [1], then turn dorsally [2], and turn again to migrate towards the midbody region [3]. Loss of *lon-2*/glypican leads to defective DTC guidance, including a failure to migrate dorsally, premature dorsal turning, or a failure to remain dorsal. (B) Quantification of the DTC migration defects in *lon-2*/glypican mutants and rescue by *lon-2(+)* (see S7 Table). For each transgenic line, transgenic animals were compared to nontransgenic sibling controls. Complete loss of *lon-2*/glypican does not enhance the defects of the *unc-6*/netrin null mutants or those of the null mutants for *unc-5*/UNC5 and *unc-40*/DCC, suggesting that *lon-2*/glypican functions in the same guidance pathway as *unc-5*/UNC5, *unc-40*/DCC, and *unc-6*/netrin (see S6 Table). (C) The axons of the GABAergic motorneurons project dorsally from the ventral midline towards the dorsal nerve cord. *unc-6*/netrin, *unc-*5/UNC5, and unc-*40*/DCC are required for this dorsal guidance of GABAergic axons. Complete loss of *lon-2*/glypican does not enhance the partially penetrant defects of *unc-40*/DCC null mutants, suggesting that *lon-2*/glypican functions in the same pathway as *unc-40*/DCC and *unc-6*/netrin to guide axons dorsally (see S5 Table). Error bars are standard error of the proportion. Asterisks denote significant difference: *** *p* ≤ 0.001 and * *p* ≤ 0.05 (*z*-tests, *p*-values were corrected by multiplying by the number of comparisons). ns, not significant.

In addition to *unc-6*/netrin acting as an attractive cue for cells expressing the *unc-40*/DCC receptor in ventral guidance, *unc-6*/netrin also acts as a repulsive cue for cells expressing both the *unc-5*/UNC5 and *unc-40*/DCC receptors, which together mediate dorsal guidance away from *unc-6*/netrin [4–6]. To address whether *lon-2*/glypican functions in *unc-6*/netrin-mediated repulsive guidance as well, we examined the dorsal migration of the distal tip cells (DTCs) and of the GABAergic motorneuron axons [4,11]. We found that *lon-2*/glypican single null mutants are defective in dorsal DTC migrations (Fig 2A and 2B) and that the complete loss of *lon-2*/glypican did not enhance the dorsal DTC migration defects of *unc-6*/netrin, *unc-40*/DCC, or *unc-*5/UNC5 null mutants (Fig 2B), indicating that *lon-2*/glypican functions in the *unc-6*/netrin-repulsive guidance pathway as well. Similarly, complete loss of *lon-2*/glypican did not enhance the defects of *unc-40*/DCC mutants in the dorsal guidance of motorneuron axons (Fig 2C). Given that loss of *lon-2*/glypican enhances the motorneuron axon guidance defects of *sdn-1* mutants as shown in [17], *lon-2/*glypican plays a role in the dorsal guidance of motorneuron axons. The lack of enhancement of the defects in the dorsal guidance of motorneuron axons of *unc-40*/DCC mutants by loss of *lon-2*/glypican further supports that *lon-2*/glypican functions in the *unc-6*/netrin pathway mediating dorsal guidance. Thus, *lon-2*/glypican may modulate *unc-6*/netrin signaling not only during attractive guidance but also during repulsive guidance.

### *unc-6*/Netrin Signaling via the *unc-5*/UNC5 Receptor Requires *lon-2*/Glypican

**Fig 3:**
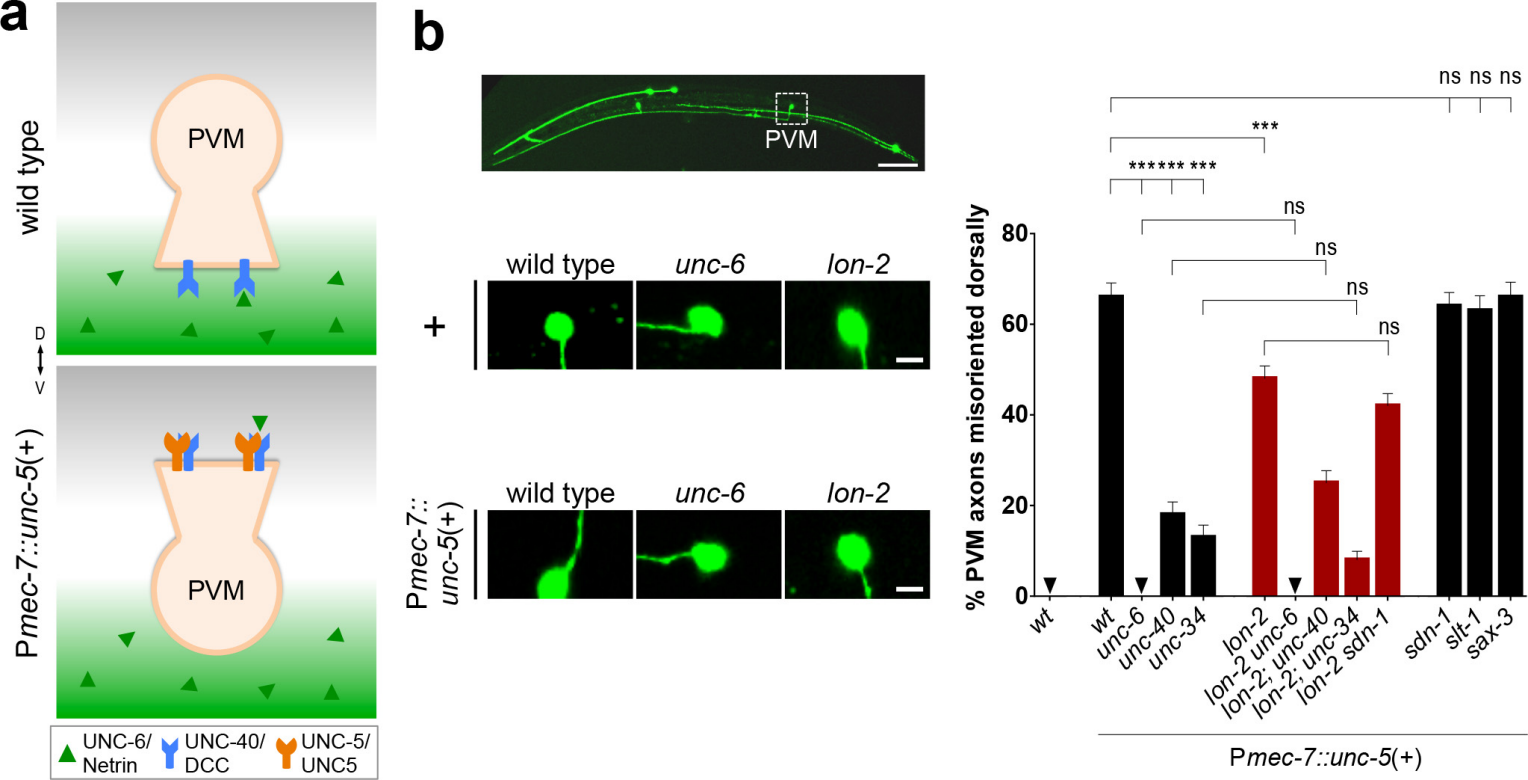
*unc-6*/netrin signaling via the *unc-5*/UNC5 receptor requires *lon-2*/glypican. (A) The axon of PVM normally migrates ventrally in the wild type, but it can be forced to migrate dorsally by misexpressing the repulsive receptor *unc-5*/UNC5. We quantified PVM since AVM could not be reliably identified (both AVM and neighboring ALMR axons project dorsally in P*mec-7*::*unc-5* transgenic animals.) (B) Upon misexpression of *unc-5*/UNC5 in PVM, using the transgene P*mec-7*::*unc-5*, the axon of PVM projects dorsally in an *unc-6*/netrin-, *unc-40*/DCC-, and *unc-34/*enabled-dependent manner. Loss of *lon-2*/glypican partially suppresses this forced dorsal migration, indicating that *unc-6*/netrin signaling depends on *lon-2*/glypican. Scale bar, 5 μm. Error bars are standard error of the proportion. Asterisks denote significant difference: *** *p* ≤ 0.001 (z-tests, *p*-values were corrected by multiplying by the number of comparisons). ns, not significant. Wild type (without *evIs25*) is the same as in Fig 1B.

To complement the above loss-of-function approach, we next used a gain-of-function strategy to test the model that *lon-2*/glypican functions in the *unc-6*/netrin signaling pathway. We focused on the axon of the PVM neuron instead of AVM, because it could reliably be identified (AVM cannot be distinguished from ALMR in these experiments). In wild-type animals, PVM, like AVM, expresses the receptor *unc-40*/DCC, and its axon grows ventrally towards *unc-6*/netrin (Fig 3A). In mutants lacking *unc-6*/netrin signaling, PVM axons that fail to extend ventrally instead extend anteriorly (never dorsally, see S4 Table). The PVM axon normally does not express the receptor *unc-5*/UNC5 that mediates repulsive guidance away from ventral *unc-6*/netrin [6], but misexpression of the receptor *unc-5*/UNC5 (using transgene P*mec-7*::*unc-5* [21]) in PVM forces its axon to extend dorsally in an *unc-6*/netrin- and *unc-40/*DCC-dependent manner (Fig 3A and 3B, [21]). We used this *unc-6*/netrin-dependent *unc-5*/UNC5-mediated abnormal dorsal migration to further investigate the function of *lon-2*/glypican in netrin signaling. By analyzing *lon-2*/glypican mutants carrying P*mec-7*::*unc-5*, we found that compete loss of *lon-2*/glypican function significantly suppressed the *unc-6*/netrin-dependent *unc-5*-mediated abnormal dorsal migration of the PVM axon, indicating that *unc-6*/netrin signaling is *lon-2*/glypican dependent (Fig 3B). In contrast, the complete loss of *sdn-1*/syndecan, of *slt-1*/Slit, or of *sax-3*/Robo function did not suppress these PVM abnormal dorsal migrations (Fig 3B, see S4 Table), highlighting the specificity of *lon-2*/glypican action on *unc-6*/netrin signaling. As expected, *lon-2 sdn-1* double mutants lacking both *lon-2*/glypican and *sdn-1*/syndecan and expressing *unc-*5/UNC5 in PVM did not further suppress the abnormal *unc-5/*UNC-5-mediated dorsal migration of PVM as compared to *lon-2* single mutants, further supporting the specificity of *lon-2*/glypican on *unc-6*/netrin signaling.

To investigate whether *lon-2/*glypican functions in the same genetic pathway as known downstream mediators of *unc-6/*netrin signaling, we tested for genetic interactions between *lon-2*/glypican and *unc-34*/enabled. *unc-34*/enabled is a regulator of actin polymerization for axonal filopodia outgrowth [18,22–26], and its role in both *unc-6/*netrin and *slt-1*/*S*lit guidance pathways renders the analysis of genetic interactions in the context of normal AVM axon guidance challenging. Therefore, we used the *unc-6*/netrin-specific gain-of-function approach as above, in which the dorsal migration of the PVM axon upon ectopic expression of *unc-5*/UNC5 is *unc-34*/enabled dependent (Fig 3B, [21,27]). We asked whether loss of *lon-2*/glypican could enhance the extent of suppression of PVM dorsal migration induced by loss of *unc-34*/enabled. We found that the PVM dorsal migration was suppressed to the same degree in the double null mutants *lon-2; unc-34* and the single mutant *unc-34*/enabled upon expression of *unc-5*/UNC5 in PVM (P*mec-7*::*unc-5*, Fig 3B). These results support that *lon-2*/glypican functions with *unc-6*/netrin and *unc-34*/enabled during axon guidance.

### Epidermal *lon-2*/Glypican Functions in Axon Guidance

**Fig 4:**
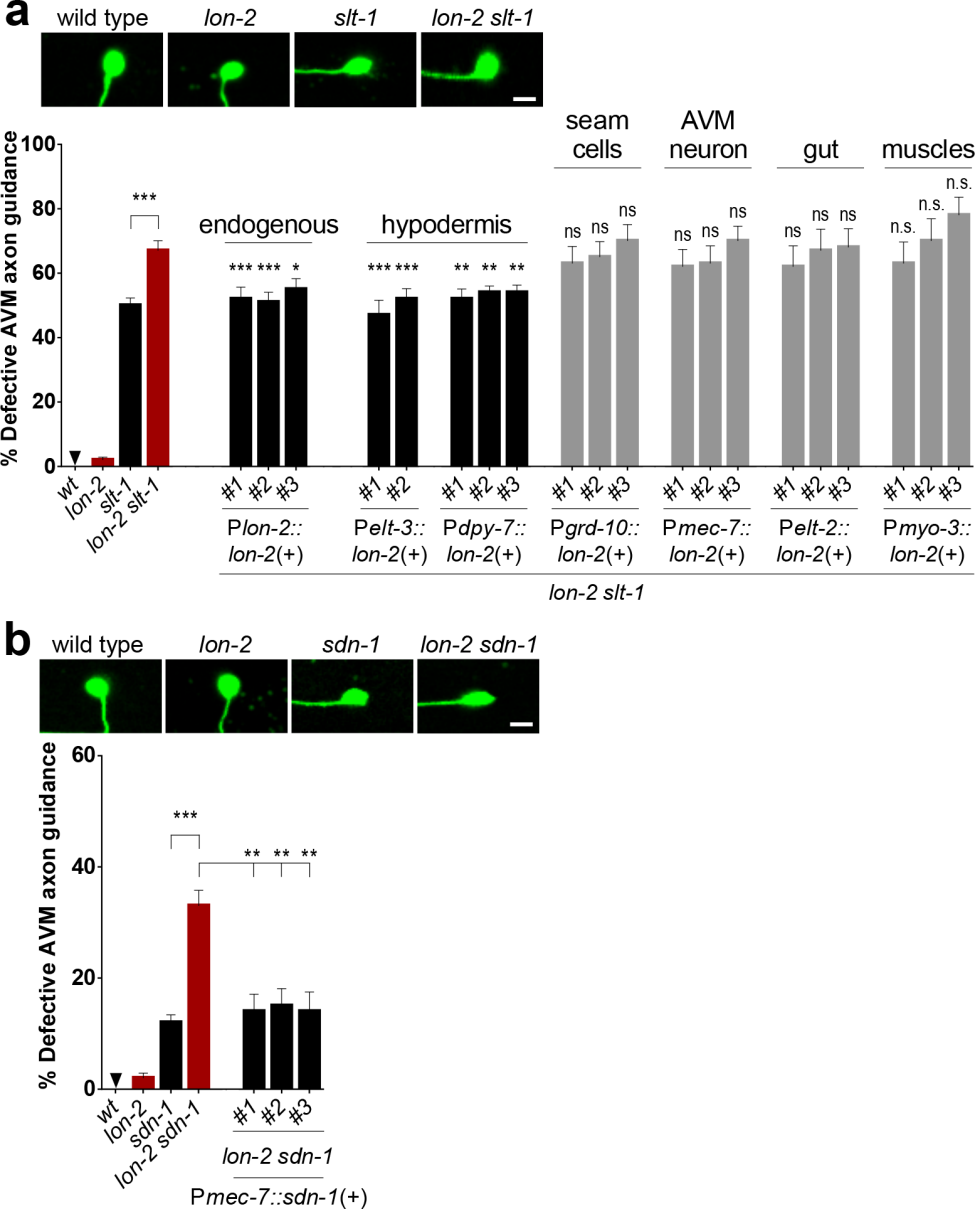
*lon-2*/glypican functions in the epidermal cells underlying the developing axon. (A) Epidermal expression of *lon-2*/glypican is sufficient for function. Providing wild-type *lon-2(+)* in the hypodermis (under the heterologous hypodermal promoters P*dpy-7* and P*elt-3*) rescues the function of *lon-2* in the double mutants *lon-2 slt-1*, as it brings the defects down to the level of *slt-1* single mutants. In contrast, expression of *lon-2*(+) in other epidermal cells (seam cells), the migrating neuron AVM, the intestine, or the body wall muscles fails to rescue the function of *lon-2*. For each rescued transgenic line, transgenic animals were compared to nontransgenic sibling controls (see S2 and S3 Tables). Data for wild type, *lon-2*, *slt-1*, and *lon-2 slt-1* are the same as in Fig 1B and 1C. (B) Expression of *sdn-1*/syndecan in the migrating neuron is sufficient for function. Providing wild-type copies of *sdn-1(+)* in AVM (expressed under the heterologous promoter P*mec-*7) rescues the axon guidance function of *sdn-1* in a *lon-2 sdn-1* double mutant. We assayed rescue of *sdn-1* function using the double mutant *lon-2 sdn-1* since it is easier to rescue defects that are 33% penetrant (as in the double *lon-2(e678) sdn-1(zh20)*) than to rescue defects that are 12% penetrant (as in the single mutant *sdn-1(zh20)*). For each transgenic line, transgenic animals were compared to nontransgenic sibling controls (see S2 Table). Data for wild type, *lon-2*, *sdn-1*, and *lon-2 sdn-1* are the same as in Fig 1B–1D. Scale bar, 5 μm. Error bars are standard error of the proportion. Asterisks denote significant difference: *** *p* ≤ 0.001, ** *p* ≤ 0.01 and * *p* ≤ 0.05 (*z*-tests, *p*-values were corrected by multiplying by the number of comparisons). ns, not significant.

The AVM growth cone extends along a basement membrane that is located between the epidermis, which is referred to as the hypodermis, and body wall muscles [11]. *lon-2*/glypican is expressed in the hypodermis and the intestine [28]. We asked in which cell type *lon-2*/glypican needs to be produced to guide AVM. We found that wild-type *lon-2(+)* transgenes expressed under the heterologous epidermal promoters P*dpy-7* and P*elt-3* (that drive expression in the hypodermis underlying the AVM growth cone, hyp7) rescued *lon-2 slt-1* double mutants back to *slt-1* single mutant levels, as efficiently as when expressed under the endogenous promoter P*lon-2* (Fig 4A, S3 Table). Rescue was not observed when we expressed *lon-2*/glypican in other epidermal cells (seam cells, P*grd-10*), in the migrating neuron itself (P*mec-7*), in the intestine (P*elt-2*), or in body wall muscles (P*myo-3*) (Fig 4A, S3 Table). Our results suggest that *lon-2*/glypican is produced by the hypodermis underlying the growth cone of AVM to function in axon guidance.

### *sdn-1*/Syndecan Functions Cell Autonomously

We found that expressing wild-type copies of *sdn-1(+)* in the AVM neuron (using the heterologous promoter P*mec-7*) rescued axon defects of *lon-2 sdn-1* double mutants (Fig 4B). Accordingly, our examination of a transgene reporting *sdn-1*/syndecan expression (*sdn-1*::*gfp* [16]) revealed that *sdn-1*/syndecan is indeed expressed in the AVM neuron (S4 Fig), at the time of its ventral migration during the first larval stage. Thus, *sdn-1*/syndecan appears to function in the migrating neuron in the *slt-1*/Slit-*sax-3*/Robo guidance pathway, whereas *lon-2*/glypican appears to function nonautonomously, as it is produced by the hypodermis underlying the migrating neuron to modulate the *unc-6*/netrin guidance pathway. Consistent with this, we found that *sdn-1(+)* cannot replace the function of *lon-2*/glypican; expressing *sdn-1*/syndecan in either the cells that normally express *lon-2*/glypican (using P*lon-2*::*sdn-1*) or the migrating neuron itself (P*mec-7*::*sdn-1*) did not rescue the loss of *lon-2*/glypican (S5 Fig), supporting that *lon-2*/glypican and *sdn-1*/syndecan have specific roles in axon guidance.

### LON-2/Glypican Lacking Its Heparan Sulfate Chain Attachment Sites Functions in Axon Guidance

**Fig 5:**
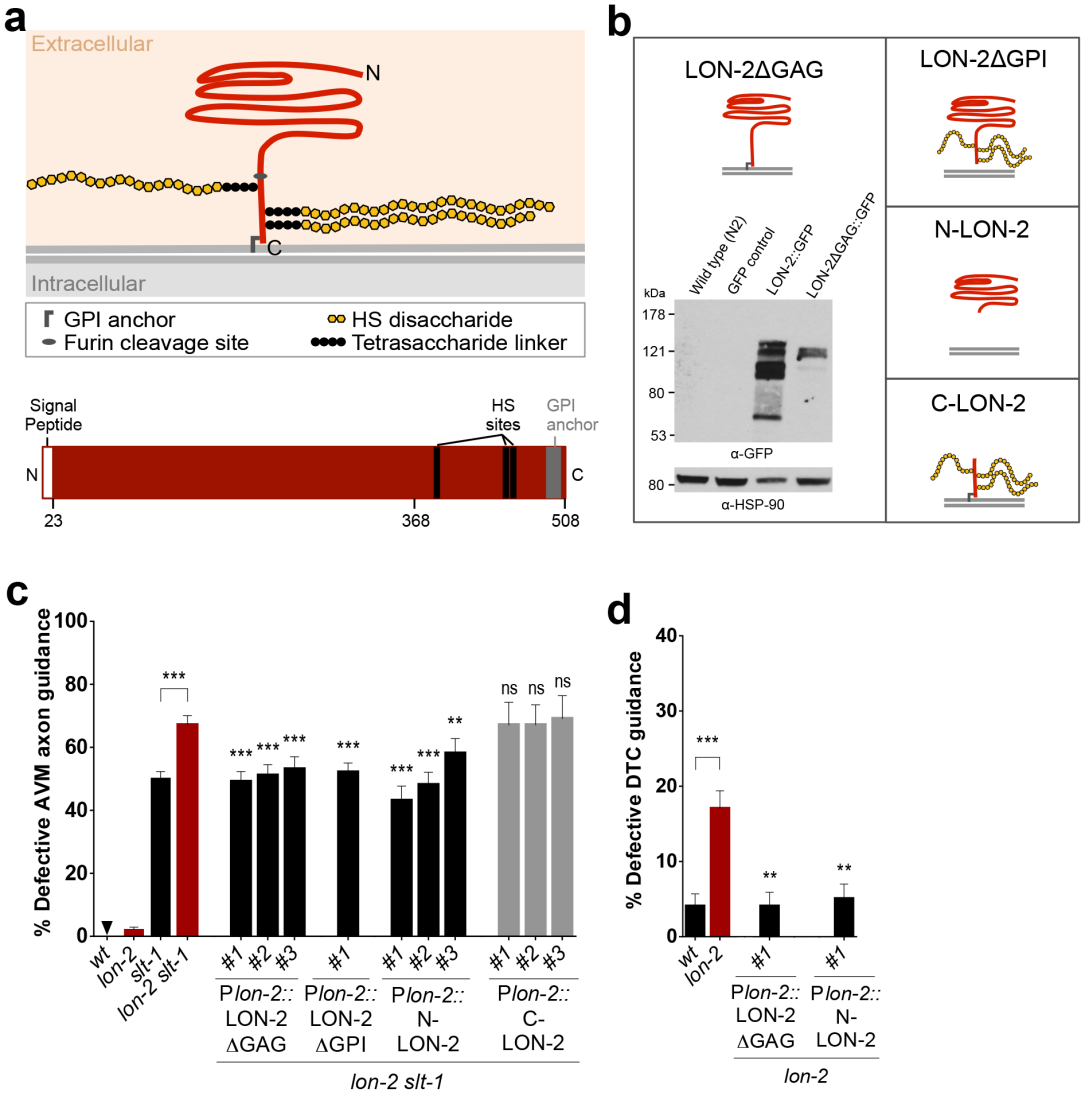
A secreted form of LON-2/glypican that lacks the heparan sulfate chain attachments is functional in axon guidance. (A) The HSPG LON-2/glypican is composed of a core protein and three HS chains. The core protein is predicted to fold into a globular domain on its N-terminal region and to be GPI-anchored. (B) Schematics of the engineered forms of LON-2 that we used: LON-2ΔGAG, in which the HS chain attachment sites are mutated; LON-2ΔGPI, in which the GPI anchor is deleted; N-LON-2, in which the C-terminus is deleted; and C-LON-2, in which the N-terminal globular domain is deleted. Western blot analysis of protein extracts of worms expressing LON-2::GFP or LON-2ΔGAG::GFP confirms that deleting the HS attachment sites on LON-2 affects HS addition on LON-2. Protein extracts from wild type (N2) and an unrelated GFP strain (*lqIs4*) are negative controls. (C) A form of LON-2/glypican lacking HS chain attachment sites (LON-2ΔGAG) functions in axon guidance. LON-2ΔGAG rescues the AVM guidance defects of double mutants *lon-2 slt-1* back to the level of *slt-1* single mutants. Secreted globular LON-2/glypican is functional in axon guidance. LON-2/glypican was engineered to be secreted by deleting its GPI anchor (LON-2ΔGPI) or by deleting the C-terminus, thus lacking the GPI anchor and the HS attachment sites (N-LON-2). Both LON-2ΔGPI and N-LON-2 function in axon guidance, as assayed by their ability to rescue axon guidance defects of *lon-2 slt-1* back down to the level of *slt-1* single mutants. In contrast, a form of LON-2/glypican containing its C-terminus including the three HS attachment sites, but lacking its N-terminal globular domain (C-LON-2), is not functional (see S2 and S3 Tables), indicating that the N-terminal globular domain of the core protein is key to the function of LON-2/glypican in axon guidance. For each rescued transgenic line, transgenic animals were compared to nontransgenic sibling controls (see S2 and S3 Tables). Data for wild type, *lon-2*, *slt-1*, and *lon-2 slt-1* are the same as in Fig 1B and 1C. (D) A form of LON-2/glypican lacking HS chain attachment sites is functional in DTC guidance. The DTC migration of *lon-2* mutants carrying the transgene P*lon-2*::LON-2ΔGAG is rescued back to wild-type levels. Secreted N-terminus globular LON-2/glypican (N-LON-2) is functional in DTC guidance, as DTC guidance defects of *lon-2* mutants are rescued by N-LON-2. Transgenic animals were compared to nontransgenic sibling controls (see S7 Table). Data for wild type and *lon-2* are the same as in Fig 2B. Error bars are standard error of the proportion. Asterisks denote significant difference: *** *p* ≤ 0.001, ** *p* ≤ 0.01 (*z*-tests, *p*-values were corrected by multiplying by the number of comparisons).

Glypicans are composed of a core protein moiety with covalently linked HS chains attached via a tetrasaccharide linker at specific Serine residues (Fig 5A, [15]). Prior studies on the role of HSPGs in other developmental pathways indicate that both the identity of the HSPG core proteins and the heterogeneity of their HS chains modified by epimerization and sulfations [15] contribute to the specificity of the interactions between particular HSPGs and the proteins that they bind [15,29,30].

To address the importance of the HS chains linked to LON-2/glypican during axon guidance, we tested whether a mutated form of LON-2/glypican lacking its HS chains could still function in axon guidance. For this experiment, the three Serine residues serving as HS chain attachment sites were mutated to Alanine residues, generating the mutant LON-2ΔGAG [31]. Western blot analysis confirmed that LON-2ΔGAG severely reduced HS chains associated with LON-2, in both worms and S2 cells (Fig 5B and S6 Fig). We then expressed LON-2ΔGAG under the P*lon-2* endogenous promoter and found that the AVM guidance defects of *lon-2 slt-1* double mutants were rescued back to the level of *slt-1* single mutants (Fig 5C). Similarly, the DTC migration defects of *lon-2*/glypican mutants were rescued by LON-2ΔGAG expression (Fig 5D). Our results indicate that LON-2/glypican devoid of its HS-chain attachment sites can function in *unc-6*/netrin-mediated guidance, suggesting that the core protein is the critical part of LON-2/glypican for its function in *unc-6*/netrin-mediated guidance of cell and axon migrations.

### LON-2/Glypican Associates with Cells Expressing UNC-40/DCC

**Fig 6:**
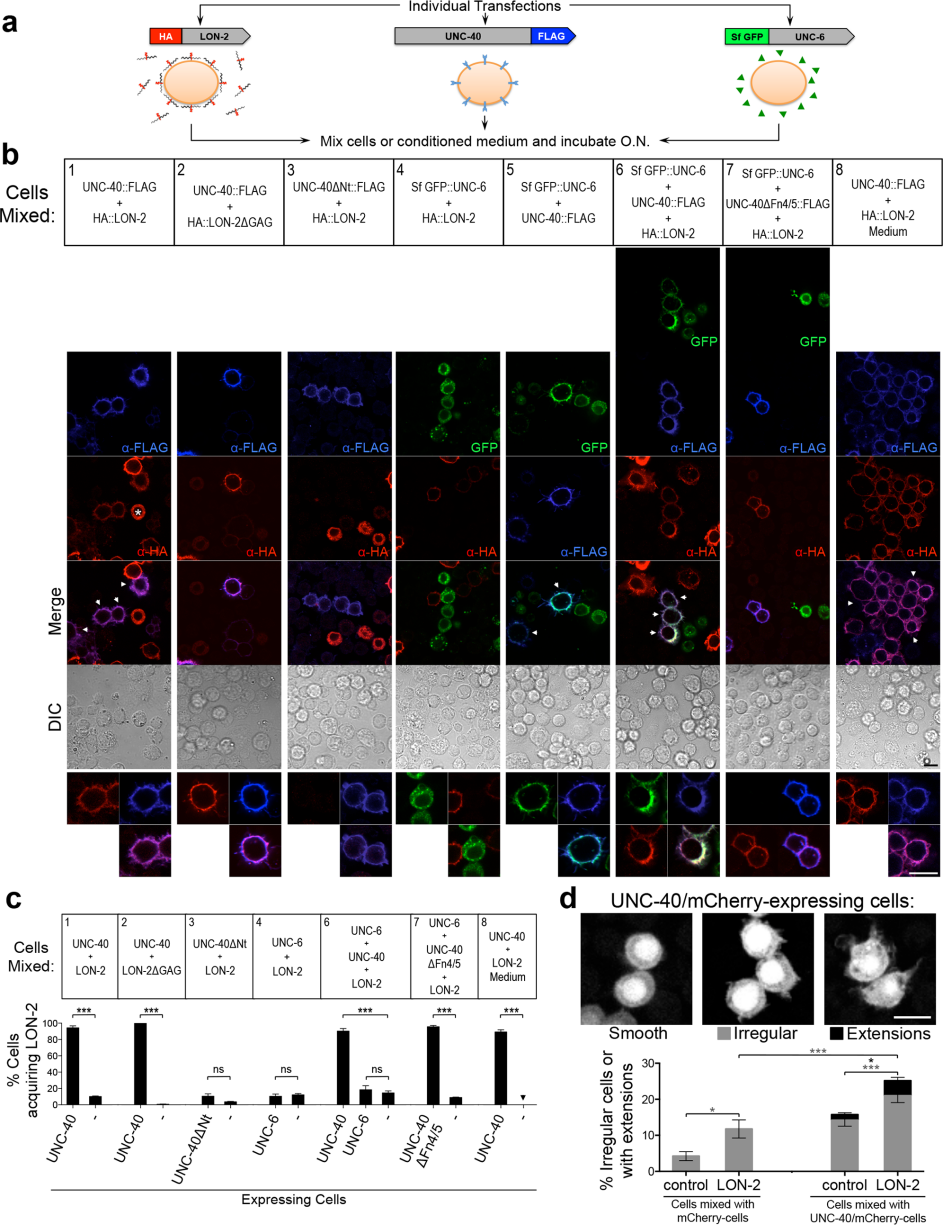
LON-2/glypican associates with UNC-40/DCC-expressing cells. (A) Experimental design. Each construct was individually and transiently transfected in S2 cells. After 2 d, cells from independent single transfections were mixed and incubated overnight and then immunostained for the corresponding tags. HA::LON-2-conditioned medium was mixed with UNC-40::FLAG-expressing cells. (B) HA::LON-2 is released from cells that produce it and associates with UNC-40-expressing cells. HA::LON-2 fills the cytoplasm of the cells that produce it (indicated by an asterisk, see also S8 Fig). Notably, HA::LON-2 is observed decorating the outline of UNC-40::FLAG-expressing cells (experiments 1, 6, 7, and 8). HA::LON-2ΔGAG also associates with UNC-40::FLAG-expressing cells (experiment 2). Cells expressing UNC-40ΔNt::FLAG that lacks the extracellular domain do not have HA::LON-2 signal, indicating that the association of LON-2 with UNC-40-expressing cells requires the extracellular domain of UNC-40 (experiment 3). HA::LON-2-conditioned medium contains HA::LON-2 that associates with UNC-40::FLAG-expressing cells, indicating that HA::LON-2 is released from the cells that produce into a diffusible form that interacts with UNC-40::FLAG cells (experiment 8). HA::LON-2 does not associate with cells expressing SfGFP::UNC-6 (experiments 4, 6, and 7) or with untransfected cells. UNC-40-FLAG-expressing cells can simultaneously associate with HA::LON-2 and SfGFP::UNC-6 (experiment 6). HA::LON-2 associates with cells expressing a mutant form of UNC-40/DCC that is unable to bind SfGFP::UNC-6, as it lacks the Fn4/5 UNC-6 binding domains (UNC-40ΔFn4/5::FLAG; experiment 7). Scale bars, 10 μm. (C) Quantification of the association of HA::LON-2 (from expressing cells, from medium of expressing cells, or from cells expressing HA::LON-2ΔGAG) with cells expressing UNC-40::FLAG, UNC-40ΔNt::FLAG, SfGFP::UNC-6, or UNC-40ΔFn4/5::FLAG and untransfected cells. Ten different optical fields containing ~300 cells from three independent experiments were quantified and averaged. (D) Cells expressing UNC-40::FLAG can display irregular morphology, which is enhanced by the presence of HA::LON-2. Images of the different morphologies displayed by UNC-40::FLAG-expressing cells: with a smooth edge, with an irregular edge, or with membrane extensions. The morphology of S2 cells expressing mCherry alone or coexpressing UNC-40::FLAG and mCherry, which were mixed with control untransfected cells or with HA::LON-2-expressing cells, were quantified for irregular edges (grey bars) or membrane extensions (black bars). A higher percentage of UNC-40::FLAG-expressing cells show membrane extensions or irregular edges when mixed with HA::LON-2-expressing cells, as compared to when they are mixed with control mCherry cells. Error bars are standard error of the mean. Asterisks denote significant difference: *** *p* ≤ 0.001, * *p* ≤ 0.05. ns, not significant. In (D), significant differences in irregular cell shape are indicated by grey asterisks, and significant difference in membrane extensions is indicated by the black asterisk.

Our above observations provide evidence that the HSPG *lon-2*/glypican functions in the same genetic pathway as *unc-6*/netrin to guide migrating axons. It has been shown in several models that HSPGs play multifaceted roles across various signaling pathways, such as facilitating ligand-receptor interactions and transporting morphogens, as well as localizing and stabilizing ligands [32,33]. We asked if the LON-2/glypican molecules might interact with either UNC-6/netrin or its receptor UNC-40/DCC, suggesting a potential mechanism of action for LON-2/glypican in *unc-6*/netrin-mediated guidance. To test these interactions, we generated epitope-tagged versions of LON-2/glypican, UNC-6/netrin, and UNC-40/DCC proteins, with human influenza hemagglutinin (HA), superfolder-GFP (SfGFP), and FLAG, respectively (Fig 6A), and used cell-mixing experiments. We independently expressed each of these labeled proteins in separate populations of *Drosophila* S2 cells for 2 d, then cocultured them overnight, and detected the tagged proteins by western blot analysis (see S7 Fig) and by immunostaining (Fig 6A).

We observed that the HA::LON-2 signal filled the cytoplasm of HA::LON-2 producing cells (indicated by white asterisks in Fig 6B experiment 1 and S8 Fig). Notably, HA::LON-2 was also found decorating the outline of UNC-40::FLAG-expressing cells (Fig 6B and 6C experiments 1, 6, 7, and 8). This observation suggests that LON-2/glypican is released from the cells that produce it, diffuses in the extracellular medium, and associates with UNC-40/DCC-expressing cells. In contrast, HA::LON-2/glypican did not bind to cells expressing SfGFP::UNC-6 (Fig 6B and 6C experiments 4, 6, and 7) or to cells expressing an unrelated type I transmembrane receptor, Evi (see S9 Fig), or to untransfected cells (Fig 6B and 6C experiments 1–8). Furthermore, we found that another HSPG, SDN-1/syndecan, did not bind UNC-40-expressing cells (see S9 Fig). These findings provide evidence for a specific interaction between LON-2/glypican and UNC-40-expressing cells.

We tested whether the HS chains of LON-2/glypican were necessary for its association with UNC-40-expressing cells. We used a mutated form of LON-2/glypican lacking its three HS chain attachment sites, HA::LON-2ΔGAG (see S6 Fig, [31]). Western blot analysis confirmed that LON-2ΔGAG severely reduced HS chains associated with LON-2/glypican (S6 Fig). We found that LON-2ΔGAG associated with UNC-40/DCC-expressing cells (Fig 6B and 6C experiment 2), suggesting that the association of LON-2/glypican with UNC-40/DCC-expressing cells is HS-chain independent.

The HA::LON-2 signal outlined the UNC-40/DCC-expressing cells (Fig 6B, experiments 1, 6, 7, and 8) suggesting a potential interaction at the cell surface. To further support this idea, we asked whether LON-2/glypican would associate with cells expressing a mutated form of UNC-40/DCC that lacks the extracellular domain and contains only the intracellular and transmembrane domains (UNC-40ΔNt::FLAG). We found that HA::LON-2 did not associate with cells expressing the UNC-40ΔNt::FLAG (Fig 6B and 6C experiment 3), indicating that the extracellular domain of UNC-40/DCC is required for LON-2/glypican to associate, as would be predicted if LON-2/glypican and UNC-40/DCC interact, directly or indirectly, at the cell surface.

Interestingly, HA::LON-2 was absent from cells expressing SfGFP::UNC-6 (Fig 6B and 6C experiments 4, 6, and 7), indicating that while LON-2/glypican interacts with cells expressing UNC-40/DCC, it does not bind to UNC-6/netrin-expressing cells in this assay. Moreover, the presence of SfGFP::UNC-6 did not reduce the ability of HA::LON-2 to associate with UNC-40/DCC-expressing cells in experiments in which the three singly transfected cell populations were mixed (Fig 6B and 6C experiment 6). These results suggest that if LON-2/glypican interacted directly or indirectly with UNC-40/DCC, then the interactions of LON-2/glypican and UNC-6/netrin would occur with different regions of UNC-40/DCC. Consistent with this possibility, we found that LON-2/glypican still associated with cells expressing UNC-40ΔFn4/5::FLAG, a mutated form of UNC-40/DCC that lacks the UNC-6/netrin-binding sites (FnIII domains 4 and 5) (Fig 6B and 6C experiment 7, [34,35]). Our results indicate that for LON-2/glypican to associate with UNC-40/DCC-expressing cells, the FnIII domains 4 and 5 of UNC-40/DCC are dispensable and UNC-6/netrin does not need to be bound to UNC-40/DCC.

### LON-2/Glypican Increases Membrane Outgrowths Triggered by UNC-40/DCC

Previous work has suggested that overexpression of DCC in cells overactivates DCC downstream signaling pathways, leading to cytoskeletal rearrangements that result in increased membrane extensions and cell surface area [36]. Similarly, expression of UNC-40/DCC leads to changes in cellular morphology in our cell assays (Fig 6D). To test whether the association of LON-2/glypican with UNC-40/DCC-expressing cells results in an activation of signaling downstream of UNC-40/DCC, we examined the impact of LON-2/glypican on the morphology of UNC-40/DCC-expressing cells. For these experiments, we mixed mCherry-expressing cells with either untransfected control cells or LON-2/glypican-expressing cells, and we also mixed UNC-40/mCherry-expressing cells with either untransfected control cells or LON-2/glypican-expressing cells. Examination of the morphology of these cells 1 d after mixing revealed that UNC-40/mCherry-expressing cells mixed with LON-2/glypican exhibited an increased frequency of irregular shapes and membrane extensions, compared to UNC-40/mCherry cells mixed with control cells (Fig 6D). Thus, consistent with a model in which LON-2/glypican functions in the UNC-6/netrin signaling pathway to guide developing axons, the association of LON-2/glypican with UNC-40/DCC-expressing cells leads to increased membrane extensions, suggestive of increased signaling downstream of the UNC-40/DCC receptor.

### LON-2/Glypican Is Released Extracellularly and Its N-terminal Domain Is Functional

While LON-2/glypican possesses a signature GPI anchor that mediates its attachment to plasma membranes (Fig 5A), our experiments indicate that LON-2/glypican is released into the extracellular milieu through cleavage where it can diffuse to associate with UNC-40/DCC-expressing cells. This is consistent with prior work demonstrating that many glypicans are shed or cleaved into a soluble form [37]. To verify that LON-2/glypican is indeed released into the extracellular medium, we collected cell-free media from HA::LON-2 cultures (HA::LON-2-conditioned medium) and added it to cells expressing UNC-40::FLAG. We found that HA::LON-2-conditioned medium contained HA::LON-2 that associated with UNC-40::FLAG-expressing cells. As above, this interaction was specific, as no HA::LON-2 signal was found on adjacent untransfected cells (Fig 6B and 6C experiment 8). This result provides compelling evidence that LON-2/glypican can be released from the membrane of LON-2/glypican-expressing cells, diffuses, and associates with UNC-40/DCC-expressing cells. We propose that using a similar mechanism, LON-2/glypican may be shed from epidermal cells and may interact with migrating axons that express UNC-40/DCC. This is consistent with our finding that LON-2/glypican is produced by the hypodermis to function nonautonomously in *unc-6*/netrin-mediated AVM axon guidance.

**Fig 7:**
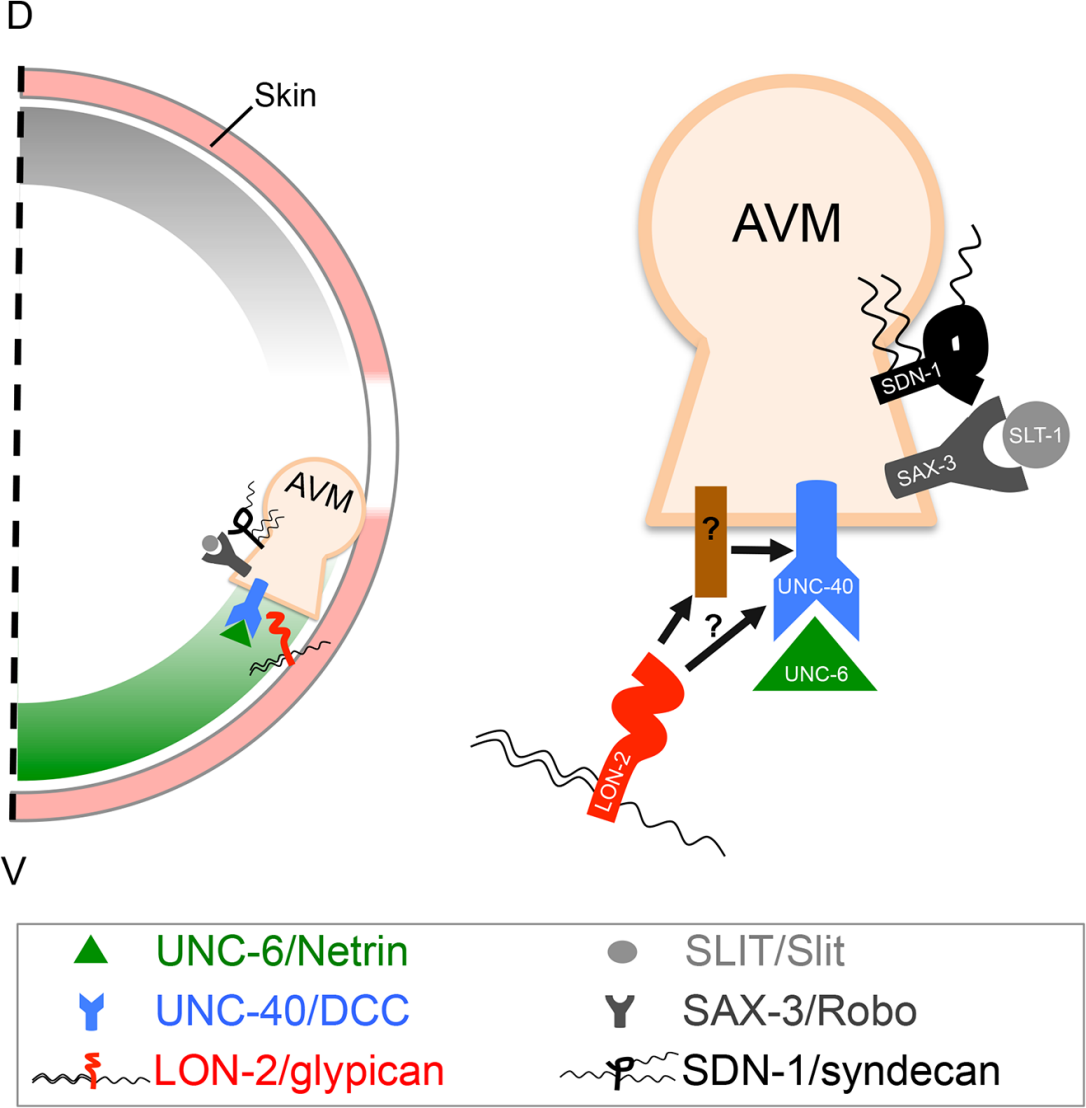
A model for the role of LON-2/glypican in UNC-6/netrin-UNC-40/DCC-mediated axon guidance. HSPG LON-2/glypican (red) is expressed from the hyp7 epidermal cells (pink) underlying the migrating growth cone of the AVM neuron (tan). LON-2/glypican is released from the hypodermal cell surface and may associate with the developing axon expressing the receptor UNC-40/DCC (blue), directly or indirectly interacting with UNC-40/DCC, to modulate UNC-6/netrin (green) signaling. A second HSPG, SDN-1/syndecan (black), acts in the SLT-1/Slit-SAX-3/Robo (grey) axon guidance pathway.

To provide evidence for the model that LON-2/glypican can function in axon guidance when detached from the plasma membrane, we used a form of LON-2/glypican lacking the GPI anchor, LON-2ΔGPI, which should be directly secreted into the extracellular milieu [31]. LON-2ΔGPI rescued the AVM guidance defects of *lon-2 slt-1* double mutants back to the level of *slt-1* single mutants (Fig 5C). We also used a truncated form of LON-2/glypican (N-LON-2) containing the N-terminal globular domain, but lacking the C-terminal region, thus removing the three HS attachment sites and the GPI membrane anchor. N-LON-2 also rescued the AVM guidance defects of *lon-2 slt-1* double mutants back to the level of *slt-1* single mutants (Fig 5C). In contrast, a reciprocal construct containing only the C-terminus with the three HS attachment sites and the GPI anchor (C-LON-2) did not rescue the AVM axon guidance defects of *lon-2 slt-1*, consistent with the model that the N-terminal globular domain of LON-2/glypican is the key functional domain during guidance (Fig 5C). A secreted form of LON-2/glypican is also functional in DTC guidance, as we found that DTC guidance defects of *lon-2*/glypican mutants could be rescued by expression of N-LON-2, containing only the N-terminal globular domain (Fig 5D). These findings also support the hypothesis that LON-2/glypican may normally be released from the hypodermis to interact with the *unc-6*/netrin pathway to direct the migrating growth cone during development (Fig 7).

## Discussion

Growth cone responses to guidance cues require precise regulation as developing axons traverse complex extracellular environments in order to reach their targets. The mechanisms by which guidance cue signals are regulated in the extracellular milieu are still poorly understood [38]. Here, we demonstrate that the *unc-6*/netrin-*unc-40*/DCC guidance system is modulated by the HSPG *lon-2*/glypican.

### *lon-2*/Glypican Is a Component of the *unc-6*/Netrin Signaling Pathways

Our studies identify the HSPG LON-2/glypican as a component of the *unc-6*/netrin attractive and repulsive signaling pathways that guide axons during development. We show that LON-2/glypican specifically acts on *unc-6*/netrin signaling independently of *slt-1*/Slit. We demonstrate that *lon-2*/glypican functions from the hypodermis, the epidermal cells that secrete the substrate along which growth cones extend [11], and that a secreted form of LON-2/glypican, containing only its N-terminal globular region and lacking its HS chains, guides cells and axons in vivo. In addition, we provide evidence that LON-2/glypican is released from cells producing it and associates with cells expressing UNC-40/DCC receptors. Taken together, our observations support a hypothetical model in which GPI-linked LON-2/glypican is produced by substrate epidermal cells, is released into the extracellular milieu, and binds growth cones expressing UNC-40/DCC receptors to regulate attractive and repulsive responses of the growth cone to UNC-6/netrin.

The impact of *lon-2*/glypican on the *unc-6*/netrin signaling pathway is highly specific. First, loss of *lon-2*/glypican, but not of *sdn-1*/syndecan, suppresses the guidance phenotypes elicited by the gain-of-function condition in which *unc-5*/UNC5 was misexpressed. Second, the complete loss of *lon-2*/glypican does not enhance the guidance defects observed in null mutants for *unc-6*/netrin or its receptors *unc-40*/DCC and *unc-5*/UNC5, whereas it does enhance the defects of several other axon guidance mutants, including *sdn-1*/syndecan, *slt-1*/Slit, misexpressed *slt-1*/Slit (P*myo-3*::*slt-1*), *sax-3*/Robo, and *sqv-5*, suggesting that *lon-2*/glypican functions specifically in the *unc-6*/netrin pathway. Third, *sdn-1*/syndecan, cannot replace *lon-2*/glypican function, highlighting a requirement for *lon-2*/glypican that cannot be achieved by any HSPG. Given that the core protein of LON-2/glypican, devoid of its HS chains, is fully functional in guidance, the specificity of action of LON-2/glypican in netrin-mediated guidance appears to reside in the core protein itself. As a note, whereas *lon-2*/glypican mutants are defective in DTC migration, the *lon-2*/glypican mutant by itself does not show drastic alterations in AVM axon guidance as is observed with other modulators [32]. It is possible that in the absence of *lon-2*/glypican, another HSPG may provide compensation or that our scoring of strong alterations in pathfinding did not include more subtle phenotypes, as could be expected from a modulator of the signal [32].

### The Core Protein of LON-2/Glypican Is Functional in UNC-6/Netrin-Mediated Guidance

We show that the LON-2/glypican core protein, devoid of HS attachment sites, is able to associate with UNC-40-expressing cells and is functional in *unc-6*/netrin-mediated guidance. Thus, the core protein is the critical region of LON-2/glypican for netrin-mediated axon guidance. This is in line with previous studies showing a contextual dependence of HS chains for glypican function. For instance, the core protein of *C*. *elegans* LON-2/glypican and of *Drosophila* glypican Dally do not require HS chains to function in the transforming growth factor beta (TGFβ) pathway [31,39]. Similarly, *Drosophila* glypican Dally-like interacts with Wg and Hh through their protein core in a HS-independent manner [33,40,41], and mammalian Glypican-3 does not require HS chains for its role in Wnt and Hh signaling [42–45]. While the HS chains are not critical for the role of LON-2/glypican in guidance, a contribution of HS chains to modulate functionality, as observed for other glypicans in the context of BMP4, Wnt3, Wg, and Hh signaling [33,39,41,42], cannot be ruled out. For instance, it is conceivable that the normal endogenous HS chains of LON-2/glypican may impact its trafficking, levels, release from the membrane, recruitment of binding partners, or recycling.

### LON-2/Glypican Is Released from the Cell Surface to Guide Axons

LON-2/glypican is predicted to localize at the cell surface via its GPI anchor [31]. However, in our cell culture studies, we demonstrate that LON-2/glypican can be released as a soluble molecule from producing cells. We also show that two truncated forms of LON-2/glypican, LON-2ΔGPI and N-LON-2, which are no longer associated with the plasma membrane and are secreted into the extracellular milieu, can function to guide axons in vivo. This indicates that LON-2/glypican is likely released from the epidermal cells to reach the growth cone to modulate its guidance. This finding raises the question of how LON-2/glypican is released from the cell membrane and how this process might be regulated during development. The release of LON-2/glypican from the surface of cells could involve phospholipases that cleave the GPI anchor and/or proteases that cleave its extracellular domain, such as at a predicted furin-cleavage site (Fig 5A, [31]).

Glypican cleavage by lipases and proteases has been demonstrated to occur and to be functionally important in other contexts, such as in regulating fibroblast growth factor (FGF) and Wnt signaling during morphogenesis [37,46]. For instance, the *Drosophila* glypican Dally-like protein is cleaved at the GPI anchor by the lipase Notum, to negatively regulate Wnts [47]. Similarly, several mammalian glypicans, including glypican-3, are cleaved by Notum [48]. The functional importance of glypican proteolytic cleavage is illustrated by the processing of glypican-3 by a furin-like convertase to modulate Wnt signaling in zebrafish [49]. In addition, glypican-1 and glypican-4 are proteolytically cleaved to stimulate long-range FGF signaling in the *Xenopus* embryo [50] and increase the efficiency of myogenic differentiation in the presence of FGF in mammalian cells [51], respectively. Our studies show that glypican processing also functions during axon guidance.

### LON-2/Glypican Associates with the Surface of UNC-40/DCC-Expressing Cells

We demonstrate that LON-2/glypican is secreted into the extracellular medium and decorates the outline of UNC-40/DCC-expressing cells. Deleting the extracellular domain of UNC-40 (UNC-40ΔNt) abrogated the association of LON-2/glypican with UNC-40/DCC-expressing cells, indicating that LON-2/glypican may interact with UNC-40/DCC at the cell surface. The association of LON-2/glypican with UNC-40/DCC may be direct or indirect through interactions with other molecules (Fig 7). Our experiments demonstrate that UNC-6/netrin binding to UNC-40/DCC was undisturbed by the association of UNC-40/DCC with LON-2/glypican, suggesting that the possible interaction of LON-2/glypican with UNC-40/DCC likely involves a region of UNC-40/DCC other that the netrin binding sites. Indeed, we found that LON-2/glypican associates with UNC-40/DCC-expressing cells even when the UNC-40/DCC receptors lack the UNC-6/netrin binding domains.

We found that LON-2/glypican leads to increased irregular morphology of UNC-40/DCC-expressing cells. Ectopic expression of DCC in mammalian cells activates downstream signaling via Cdc42 and Rac1, producing cytoskeletal rearrangements that lead to filopodia outgrowth and cell surface extensions [36]. Our finding that the presence of LON-2/glypican enhances the UNC-40/DCC-induced irregular cell morphology and filopodia-like extensions suggests that the association of LON-2/glypican with UNC-40/DCC-expressing cells may increase signaling downstream of UNC-40/DCC. Consistent with this notion, we show that *lon-2*/glypican functions in the same signaling pathway as the UNC-40/DCC downstream mediator *unc-34*/enabled during axon guidance.

Our results suggest a possible regulatory mechanism in the extracellular space in which secreted LON-2/glypican modulates the activity of the receptor UNC-40/DCC. LON-2/glypican may directly bind UNC-40/DCC, or alternatively, LON-2/glypican may instead interact with other molecules to impact UNC-40/DCC to modulate its stability, distribution, or activity. Alternatively, LON-2/glypican could potentially function as a co-receptor for UNC-6/netrin, where it may facilitate the formation of UNC-6/netrin-UNC-40/DCC-LON-2/glypican signaling complexes, similar to the situation in FGF signaling [15]. It is also conceivable that LON-2/glypican could bind UNC-6/netrin directly as well, even if undetected in our assays, as netrin has been found to bind heparin in vitro [3,52,53]. Previous studies have also documented the binding of DCC to heparin in vitro [7,8], and while we have found that the core protein is the critical portion of LON-2/glypican in netrin-mediated axon guidance, it remains possible that the endogenous HS chains contribute to the function of LON-2/glypican in axon guidance.

In summary, our studies uncover a novel mechanism by which UNC-6/netrin signaling through its UNC-40/DCC receptor is modulated by the HSPG LON-2/glypican during axon pathfinding. Given the evolutionary conservation of the UNC-6/netrin pathway components (UNC-6/netrin and its receptors UNC-40/DCC and UNC-5/UNC5) and of glypicans (LON-2 is most similar to mammalian glypican-3) and that synthesis of HS chains is required for mammalian axons to respond to netrin-1 in vitro [9,10], glypicans are likely to play a role in netrin-mediated axon pathfinding in mammals as well. Our findings provide a general mechanism for the extracellular regulation of growth cone responses to netrin during the development of nervous systems.

## Materials and Methods

### Nematode Strains and Genetics

Nematode cultures were maintained at 20°C on NGM plates seeded with OP50 bacteria as described [54]. Strains were constructed using standard genetic procedures and are all listed in S8 Table. Genotypes were confirmed by genotyping PCR or by sequencing when needed, using primers listed in S9 Table.

### Real-Time PCR (RT-PCR) for *gpn-1* Alleles

Total RNA was extracted from worm samples using Trizol (Invitrogen) according to the manufacturer’s instructions. 500 ng RNA was used to reverse transcribe using the High Capacity cDNA Reverse Transcription Kit (Applied Biosystems) and random primers. PCR reactions were carried out with first-strand cDNA template, primers oCB834 (ATCAAGACCGAGTGATAGTG) and oCB1321 (TGGCGAGTATTCCCGTTTAG) were used for *gpn-1* cDNA amplification, and primers oCB992 (TCGCTTCAAATCAGTTCAGC) and oCB993 (GCGAGCATTGAACAGTGAAG) were used for the control gene Y45F10D.4 [55] cDNA amplification.

### Neuroanatomical and Distal Tip Cell Observations

Animals were mounted on agarose pads, anaesthetized with 100 mM sodium azide, and examined under a Zeiss Axio Scope.A1 or a Zeiss Axioskop 2 Plus.

#### AVM and PVM axon guidance analysis

Axons of neurons AVM and PVM were examined in L4 larvae and adult animals using *zdIs5*, an integrated P*mec-4*::*gfp* reporter [56]. Animals with the cell body of AVM posterior to the vulva (cell migration defect) were excluded from axon guidance analysis.

#### AVM analysis

Worms were counted as mutant for AVM ventral axon guidance if (a) AVM failed to send an axon ventrally and instead projected laterally to the anterior or (b) the AVM axon projected laterally, in the anterior or the posterior direction, for at least ~15 μm (>3 AVM cell body lengths) before projecting to the ventral side. The angle between the anterior/posterior axon projection and the ventral axon projection had to be >45° to be counted as mutant, therefore excluding animals with a slight curve in the ventral axon of AVM from the mutant count.

#### PVM analysis criteria

Worms were counted as having their PVM axon misoriented dorsally if the axon of PVM was projecting to the dorsal side of the animal. The vulva was used as a reference for the ventral side. Worms whose *zdIs5* labeled neurons were too misplaced to be identified were excluded from analysis.

#### Distal tip cell guidance analysis

The path of migration of the DTCs brings about the shape of the mature gonad arms (Fig 2A, [11]). In the wild type, the DTC migrates away from the vulva location along the anteroposterior axis of the animal. The DTC then turns dorsally to reach the dorsal side of the animal, where it then migrates towards the vulva along the anteroposterior axis of the animal. To infer the path of DTC migration, gonad arms were examined in late L4 and young adult animals using DIC microscopy. Animals were counted as having abnormal gonad arm shapes when (a) the distal arm of the gonad was located ventrally instead of dorsally, indicating a failure of the DTC to migrate dorsally; (b) the proximal gonad arm was too short, resulting from a premature turn of the DTC towards the dorsal side; or (c) the gonad arm was twisted over itself.

#### GABAergic motorneuron axon analysis

Commissures of the GABAergic motorneurons were analyzed in L4 larvae and adults using *ufIs34*, an integrated P*unc-47*::*mCherry* reporter [57]. For each animal, all GABAergic commissures (except DD1 and VD2) were counted and categorized as either reaching the dorsal cord or failing to reach the dorsal cord. The fraction of commissures failing to reach the dorsal cord among all the commissures extending from the ventral cord was determined for each animal, averaged for the genotype, and expressed as a percentage.

### *C*. *elegans* Constructs and Microinjections to Generate Transgenic Animals

All inserts of finalized clones were verified by sequencing.

#### P*lon-2*::*lon-2* (PCR product)

Primers oCB1070 (CATGATAAGCTTTTCAAATTGGCGGTTAACTG) and oCB1124 (ATCATGGGGCCCTAAGCTGAATTCCCATAAC) were used to amplify a PCR product out of N2 genomic DNA containing bases 13,104 of cosmid C39E6 to 26,408 of cosmid F55D10 of the *lon-2* locus.

#### P*dpy-7::lon-2* (pCB268)

Vector P*dpy-7* was cut with *Xma*I and *Apa*I and ligated with insert of *lon-2* cDNA digested out of pCB251 (P*mec-7*::*lon-2*) with *Xma*I and *Apa*I.

#### P*elt-3::lon-2* (pCB304)

Vector P*dpy-7*::*lon-*2 (pCB268) was digested with *Hind*III and *Xma*I to release P*dpy-7* and ligated with insert of P*elt-3* 2-kb promoter (coordinates on cosmid K02B9: 16,117 to 18,081) amplified out of N2 genomic DNA (modeled after [58]) using primers oCB1063 (CATGATAAGCTTTGTGACACGTTGTTTCACG) and oCB1064 (ATCATGCCCGGGGAAGTTTGAAATACCAGGTAG) to add on *Hind*III and *Xma*I sites.

#### P*grd-10::lon-2* (pCB266)

Vector P*grd-10*::*GFP* (in pPD95.75 backbone) was digested with *Kpn*I and *Eco*RI to release GFP and ligated with insert of *lon-2* cDNA (yk1346g07) amplified with primers oCB1034 (CATGATGGTACCATGGTCTTCCGGTGGCTCATTC) and oCB1035 (ATCATGGAATTCTCAAAAAAGTTTAATAACTGC) to add on *Kpn*I and *Eco*RI sites.

#### P*mec-7::lon-2* (pCB251)

Vector pPD96.41 was digested with *Xma*I and *Nhe*I and ligated with insert of *lon-2* cDNA (yk1346g07) amplified by primers oCB963 (CATGATCCCGGGATGGTCTTCCGGTGGCTCATTC) and oCB964 (ATCATGGCTAGCTCAAAAAAGTTTAATAACTGC) to add on *Xma*I and *Nhe*I sites.

#### P*elt-2::lon-2* (pCB218)

Vector P*dpy-7*::*lon-*2 (pCB268) was digested with *Hind*III and *Xma*I to release P*dpy-7* and ligated with insert of P*elt-2* 1-kb promoter (coordinates on cosmid C33D3: 2933–3875) amplified out of N2 genomic DNA (modeled after [58]) using primers oCB1059 (CATGATAAGCTTTTGATTTTGTTTCACTCTGTG) and oCB1060 (ATCATGCCCGGGTATAATCTATTTTCTAGTTTC) to add on *Hind*III and *Xma*I sites.

#### P*myo-3::lon-2* (pCB332)

Vector pCB268 (P*dpy-7*::*lon-2*) was digested with *Hind*III and *Xba*I to release P*dpy-7* and was ligated with insert of P*myo-3* digested out of vector pPD95.86 with *Hind*III and *Xba*I.

#### P*lon-2::lon-2* (pCB246)

Vector P*dpy-7*::*lon-*2 (pCB268) was digested with *Hind*III and *Xma*I to release P*dpy-7* and ligated with insert of *lon-2* 3-kb promoter (coordinates on cosmid C39E6: 13,104–10,105) amplified out of N2 genomic DNA (modeled after [28]) using primers oCB1070 (CATGATAAGCTTTTCAAATTGGCGGTTAACTG) and oCB1069 (ATCATGCCCGGGTCTGAAATTTTGAATATGTAAGC) to add on *Hind*III and *Xma*I sites.

#### P*lon-2*::LON-2ΔGPI (pCB269)

Vector pCB246 (P*lon-2*::*lon-2*) was digested with *Xma*I and *Eco*RI to release the *lon-2* cDNA and ligated with insert of *lon-2* cDNA (yk1346g07) amplified with primers oCB963 (CATGATCCCGGGATGGTCTTCCGGTGGCTCATTC) and oCB1074 (ATCATGCTCGAGTCAATCCGGCTGAATTTCTTTTTCC) to add on *Xma*I and *Eco*RI sites and generate a truncated form of LON-2 after amino acid 488 to remove the GPI anchor (modeled after [31]).

#### P*lon-2*::N-LON-2 (pCB270)

Vector pCB268 (P*dpy-7*::*lon-2*) was digested with *Xma*I and *Eco*RI to release the *lon-2* cDNA and was ligated with insert of *lon-2* cDNA (yk1346g07) amplified by primers oCB963 (CATGATCCCGGGATGGTCTTCCGGTGGCTCATTC) and oCB1075 (ATCATGGAATTCTCACCTTCCGAGTCGGTCCCACG), to add on *Xma*I and *Eco*RI sites and generate a truncated form of LON-2 after amino acid 368, i.e., containing amino acids 1–368 (modeled after [28]).

#### P*lon-2*::C-LON-2 (pCB311)

Vector pCB246 (P*lon-2*::*lon-2*) was digested with *Xma*I and *Nhe*I to release *lon-2* cDNA and was ligated with insert of *lon-2* cDNA amplified by primers oCB964 (ATCATGGCTAGCTCAAAAAAGTTTAATAACTGC) and (1) oCB1253 (TCCGTCCTACCTGCAGAAGAAGTGAAAATCTGTGATCACTCG), (2) oCB1254 (ATTCTTTTTGTATTGCTCTACCGGTCCGTCCTACCTGCAGAAG), and (3) oCB1255 (CATGATCCCGGGATGGTCTTCCGGTGGCTCATTCTTTTTGTATTGCTCTACC) to add on *Xma*I and *Nhe*I sites, to add on the 22 amino acid *lon-2* signal peptide sequence, and to truncate LON-2 to begin at amino acid 369. Thus, this construct codes for 22 amino acid residues of the signal peptide of LON-2, followed by residues 369–508.

#### P*mec-7::sdn-1* (pCB242)

Vector pPD96.41 was digested with *Xma*I and *Xho*I and ligated with insert of *sdn-1* cDNA (yk139f3) amplified with primers oCB903 (CATGATCCCGGGATGATTCTGAAACTCAATTTC) and oCB904 (ATCATGCTCGAGTTACGCGTAAAATTCTTTTG) to add on *Xma*I and *Xho*I sites.

#### P*lon-2::sdn-1* (pCB312)

Vector pCB246 (P*lon-2*::*lon-2*) was digested with *Xma*I and *Eco*RI to release *lon-2* and was ligated with an insert of *sdn-1* cDNA digested out of pCB242 (P*mec-7*::*sdn-1*) with *Xma*I and *Eco*RI.

Transgenic animals were generated by standard microinjection techniques [59]. Each construct or PCR amplicon was injected at 1, 5, 10, or 25 ng/μL with one or two coinjection markers that included pRF4 (125–150 ng/μL), P*ttx-3*::*mCherry* (50 ng/μL), P*ceh-22*::*gfp* (50–75 ng/μL), and/or P*unc-122*::*rfp* (50–75 ng/μL). pBSK+ (90–100 ng/μL) was used to increase total DNA concentration if needed. For details on specific coinjection marker(s) used for each rescued transgenic line, see S3 and S7 Tables.

### Western Blot Analysis of LON-2::GFP and LON-2ΔGAG::GFP Expressed in Worms

Mixed-stage wild-type (N2), GFP control (*lqIs4*), LON-2::GFP (TLG257), and LON-2ΔGAG::GFP (TLG199) worms were collected in buffer and protease inhibitors (Roche). Worm pellets were subjected to repeated freeze-thaw cycles. Protein concentration was measured using the Pierce 660 nm Protein Assay on a Nanodrop. 70 μg of samples mixed with 2x Laemmli sample buffer (Bio-Rad) were boiled, separated by SDS-PAGE on a 4%–20% Mini-Protean TGX gel (Bio-Rad), and transferred to PVDF membrane. Membranes were incubated in 1:3000 anti-GFP primary antibody (Millipore #AB3080) and 1:9000 goat anti-rabbit HRP secondary antibody (Bio-Rad #166-2408EDU). For the loading control, membranes were incubated in 1:5000 anti-HSP90 antibody (CST #4874) and 1:10000 goat anti-rabbit HRP secondary antibody (Bio-Rad #166-2408EDU). Signal was revealed using Clarity Western ECL Substrate (Bio-Rad) and imaged using film (LabScientific).

### Constructs to Express Variants of LON-2, UNC-40, UNC-6, SDN-1, and Evi in S2 Cells

All inserts of finalized clones were verified by sequencing.

#### HA::LON-2 (pCB285)

The *C*. *elegans lon-2* full-length cDNA (yk1346g07) was amplified by PCR with primers adding the *Eco*RI and *Apa*I restriction enzyme sites and cloned into pBlueScript II (Life Technologies). HA::LON-2 was made by synthesizing a DNA fragment, carrying the restriction sites *Eco*RI and *Hinc*II and containing the 5ʹ end of the *lon-2* cDNA with the HA tag added after the signal peptide sequence (at cDNA position 61) and cloned into pBlueScript/*lon-2*. The HA::LON-2 *Eco*RI/*Apa*I fragment was then cloned into the pActin5.1/V5-His vector (Life Technologies).

#### HA::LON-2::myc (pCB313)

The HA::LON-2::myc construct was made by synthesizing the 3ʹ end of the *lon-2* cDNA (bases 739–1527) with the myc tag DNA sequence before the GPI anchor domain sequence (at cDNA position 1375) and an *Apa*I restriction site at the 3ʹ end. The *Pml*I/*Apa*I synthesized fragment was then cloned into the *pActin5*.*1*/HA::LON-2 (pCB285) construct cut with the same restriction enzymes.

#### HA::LON-2ΔGAG (pCB295)

The LON-2ΔGAG construct was made by replacing the *Xho*I/*Bsm*I fragment (bases 803–1340 of the *lon-2* cDNA) in the pBlueScript/HA::*lon-2* plasmid with synthesized fragment where the *lon-2* sequence is mutated at the three predicted heparan sulfate chain attachment sites (S374A, S442A, and S444A) modeled after [31]. Similar to the wild-type version of *lon-2*, the HA::LON-2ΔGAG *Eco*RI/*Apa*I fragment was then cloned into the pActin5.1/V5-His vector (Life Technologies).

#### HA::LON-2ΔGAG::myc (pCB330)

The double tagged HA::LON-2ΔGAG::myc construct was made by replacing the *Bsm*I/*Apa*I fragment (bases 1340–1527 of the *lon-2* cDNA) in the pBlueScript/HA::LON-2ΔGAG plasmid with a synthesized fragment containing the myc tag DNA sequence before the GPI anchor domain sequence (at cDNA position 1375) and an *Apa*I restriction site at the 3ʹ end. The HA::LON-2ΔGAG::myc *Eco*RI/*Apa*I fragment was then cloned into the pActin5.1/V5-His vector (Life Technologies).

#### SfGFP::UNC-6 (pCB292)

The *C*. *elegans unc-6* full-length cDNA (yk603d12) was amplified by PCR with primers adding the *Eco*RI and *Apa*I restriction enzyme sites and cloned into pBlueScript II. The superfolder-GFP::UNC-6 construct was made by synthesizing a DNA fragment, carrying the restriction sites *Eco*RI and *Hinc*II, and containing the 5ʹ end of the *unc-6* cDNA with the superfolder-GFP sequence after the signal peptide sequence (cDNA position 70) and cloned into pBlueScript/*unc-6*. The superfolder-GFP::UNC-6 *Eco*RI/*Apa*I fragment was then cloned into the pActin5.1/V5-His vector.

#### UNC-40::FLAG (pCB301)

The *C*. *elegans* full length *unc-40* cDNA (yk449d8) was amplified by two sequential PCR reactions that added the *Xho*I and *Apa*I restriction enzyme sites, as well as the FLAG tag at the 3ʹ end before the stop codon (adding the FLAG tag at the C-terminus of UNC-40 after the intracellular domain). This UNC-40::FLAG cDNA was then cloned into the pActin5.1/V5-His vector.

#### UNC-40ΔNt::FLAG (pCB310)

The *unc-40* intracellular fragment (cDNA bases 3022–4245 that include the coding sequence for the transmembrane and intracellular domains of UNC-40), along with the FLAG tag, was amplified by PCR using the UNC-40::FLAG cDNA as template and primers carrying the *Eco*RI and *Apa*I sites. A start codon (ATG) was added to the forward primer. The UNC-40ΔNt::FLAG fragment was then cloned into the pActin5.1/V5-His vector.

#### UNC-40ΔFn4/5::FLAG (pCB334)

Using nested primers, the UNC-40ΔFn4/5 construct was made by PCR amplifying a 2-kb DNA fragment from genomic DNA from the strain NK821 *qyIs155*, which contains a deletion of fibronectin III domains 4 and 5 of the *unc-40* cDNA ([34]). The nested PCR added the FLAG tag to the 3ʹ end of the UNC-40ΔFn4/5 cDNA fragment. This final fragment was cloned into the pActin5.1/*unc-40* vector using *Dra*III and *Apa*I restriction enzymes, thus generating an *unc-40* full-length cDNA with a deletion of the fibronectin III domains 4 and 5.

#### SDN-1::myc (pCB336)

The *C*. *elegans sdn-1* cDNA, excluding the coding region for the transmembrane and intracellular C-terminal tail (cDNA bases 1–678), was amplified by two sequential PCR reactions adding the *Eco*RI and *Apa*I restriction sites and the myc tag sequence at the 3ʹ end before the stop codon. The *sdn-1*::myc *Eco*RI/*Apa*I fragment was then cloned into the pActin5.1/V5-His vector (Life Technologies).

The pAc/Evi::EGFP [60] and pAc/GFP constructs were a gift from V. Budnik (University of Massachusetts Medical School).

### Western Blot Analysis of HA::LON-2::myc and HA::LON-2ΔGAG::myc Expressed in S2 Cells

S2 cells were transfected with HA::LON-2::myc (pCB313) and HA::LON-2ΔGAG::myc (pCB330) constructs. Cells were washed once with 1X Phosphate Buffered Saline and lysed for 30 min at 4°C in 1X Phosphate Buffered Saline, 0.5% Triton X-100, and 1X Protease Inhibitor Cocktail (Roche). Samples of supernatant and cell lysates were each mixed with 2X Laemmli sample buffer (BioRad). Proteins were separated by SDS-PAGE and transferred to PVDF membrane. Membranes were incubated with rabbit anti-HA (Life Technologies #715500) and rabbit anti-myc (Santa Cruz #sc-789) primary antibodies and HRP-linked goat anti-rabbit (Bio-Rad #166-2408EDU) secondary antibody. Signals were revealed by chemiluminescence with Clarity Western ECL Substrate (BioRad) and imaged using the ChemiDoc System (BioRad).

### Western Blot Analysis of HA::LON-2::myc, UNC-40::FLAG and SfGFP::UNC-6 Expressed in S2 Cells

S2 cells were independently transfected with HA::LON-2::myc (pCB313), UNC-40::FLAG (pCB301), or SfGFP::UNC-6 (pCB292) constructs. 48 h after transfection, old culture medium was removed, and new medium was added to resuspend the cells. Equal volumes of resuspended cells that had been transfected with individual constructs were mixed and cocultured overnight. Cells were harvested, centrifuged, and combined with their corresponding supernatant from each of these cell mixes. 100 μL of supernatant of each mixture was saved and kept on ice. Cell pellets were washed once with 1X Phosphate Buffered Saline and lysed for 30 min at 4°C in 100 μL of ice-cold RIPA buffer (50 mM Tris HCl pH 7.5, 150 mM NaCl, 1% Triton-X100, 0.5% sodium deoxycholate, 0.1% SDS, and 1mM EDTA pH 8.0) supplemented with Protease Inhibitor Cocktail (Roche) and PMSF. Cell lysates were combined with their corresponding supernatant and mixed with 2X Laemmli sample buffer (BioRad). Each sample was split into three in order to run three protein gels in parallel. Proteins were separated by SDS-PAGE and transferred to PVDF membrane. Membranes were incubated with rabbit anti-myc (Santa Cruz #sc-789), mouse anti-FLAG (Sigma #F3165), and rabbit anti-GFP (Millipore AB3080) primary antibodies as well as HRP-linked goat anti-rabbit (Bio-Rad #166-2408EDU) and HRP-linked horse anti-mouse (Vector Labs PI-2000) secondary antibodies. Signals were revealed by chemiluminescence with Clarity Western ECL Substrate (BioRad) and imaged using the ChemiDoc System (BioRad).

### S2 Cell Culture, Transfection, Mixing, and Immunostaining

S2 cells were maintained in SFX Insect Media (HyClone) containing 10% Fetal Bovine Serum (HyClone) and Penicillin-Streptomycin (50 units-50 μg/mL) (Sigma). 70%–90% confluent S2 cells were transfected with 500 ng of each construct using Effectene (Qiagen) according to the manufacturer’s protocol. 48 h after transfection, old culture medium was removed and new medium was added to resuspend the cells. Equal volumes of resuspended cells that had been transfected with individual constructs were plated onto coverslips and cocultured overnight. Cells were then fixed with 4% paraformaldehyde and immunostained with rabbit anti-HA (Life Technologies #715500) and mouse anti-FLAG (Sigma #F3165) primary antibodies and Alexa594 donkey anti-rabbit (Life Technologies #R37119) and Alexa647 goat anti-mouse (Life Technologies #A21235) secondary antibodies. Confocal analysis was performed on a Zeiss LSM 5 Pascal confocal microscope. Confocal images were processed using ImageJ. Each experiment was repeated at least three times.

For the experiment in which we use HA::LON-2-conditioned medium (supernatant) of cells expressing HA::LON-2, the culture medium was also changed 48 h after transfection, fresh medium was added, and the cells were incubated for another 48 h. This medium was collected and centrifuged at 1,500 rpm to remove cells and debris. This supernatant was added onto cells expressing UNC-40::FLAG, incubated overnight, and as above, fixed, stained, and imaged.

### Analysis of the Shape of UNC-40-Expressing Cells

Independent populations of S2 cells were transfected with (1) 450 ng of pActin5.1::mCherry alone, (2) 50 ng of the UNC-40::FLAG construct plus 450 ng of the cotransfection marker pActin5.1::mCherry, or (3) 500 ng of HA::LON-2. The medium was changed and cells were mixed 48 h after transfection. Control mCherry-expressing cells were mixed with untransfected cells or with HA::LON-2-expressing cells. Similarly, UNC-40::FLAG/mCherry-expressing cells were mixed with untransfected cells or with HA::LON-2-expressing cells. To maintain the total number of cells constant in our different mixes, one volume of UNC-40::FLAG/mCherry cells was mixed with either (a) one volume of control/untransfected cells or (b) one volume of LON-2-transfected cells. Cell mixes were cocultured overnight. Cells were then fixed with 4% paraformaldehyde and examined under a Zeiss LSM 5 Pascal confocal microscope. Control mCherry-expressing cells or UNC-40::FLAG/mCherry-expressing cells were identified by the cotransfection marker mCherry. 20 fields of ~300 cells each per mix per were photographed for each of three independent experiments. Cells were categorized as having the typical S2 cell round and smooth shape, irregular edges, and/or extensions protruding from the cell.

## Supporting Information

S1 Data Excel file containing, in separate sheets, the underlying numerical data for the graphs in Figs 1B, 1C, 1D, 1E, 2B, 2C, 3B, 4A, 4B, 5C and 5D and S1, S3 and S5 Figs. (XLSX)

S2 Data Excel file containing, in separate sheets, the underlying numerical data for the graphs in Fig 6C and 6D and S9B and S9C Fig. (XLSX)

S1 Fig Ventral guidance of the AVM axon. Mutations in *unc-6*/netrin and *slt-1*/slit pathways result in partially redundant defects, as previously established by the Culotti and Bargmann labs [13]. Mutants displayed here never exhibit a dorsally migrated AVM axon. Error bars are standard error of the proportion. Asterisks denote significant difference: *** *p* ≤ 0.001 (*z*-tests, *p*-values were corrected by multiplying by the number of comparisons). ns, not significant.
(TIF)

S2 Fig *gpn-1(ok377)* and *gpn-1(tm595)* are likely null alleles. (A) *gpn-1(ok377)* and *gpn-1(tm595)* are deletions (brackets) in the *gpn-1* locus. (B) RT-PCR using primers oCB834 and oCB1321 yields truncated products in *gpn-1(ok377)* and *gpn-1(tm595)*. Y45F10D.4 is a housekeeping gene used as an RT-PCR control [55]. Sequencing of the *gpn-1* RT-PCR products for *ok377* (blue) reveals that the transcript lacks most of exon 3 and has several in-frame stop codons (*), as intronic sequence (hatch pattern) gets incorporated into the mature transcript. Sequencing of the *gpn-1* RT-PCR product for *tm595* (red) reveals that the transcript lacks exons 2 and 3 and has several in-frame stop codons (*), as intronic sequence (hatch pattern) gets incorporated into the mature transcript. No alternatively spliced products were detected in the mutants *ok377* and *tm595*. (C) *gpn-1(ok377)* and *gpn-1(tm595)* are strong loss-of-function mutations, likely nulls, in which, at most, small truncated proteins would get produced.
(TIF)

S3 Fig Loss of *lon-2* enhances AVM guidance defects of *sqv-5*. Loss of function of *sqv-5*, the gene coding for the chondroitin sulfate polymerase [61], leads to defective AVM ventral axon guidance, which is significantly enhanced by loss of *lon-2* function. Error bars are standard error of the proportion. Asterisks denote significance: *** *p* ≤ 0.001 (*z*-tests, *p*-values were corrected by multiplying by the number of comparisons). (see also S2 Table).
(TIF)

S4 Fig SDN-1::GFP expression in the neuron AVM. Using the translational fusion *sdn-1*::*gfp (opIs171)*, we found that SDN-1::GFP is expressed in hypodermal cells and neurons, as previously reported [16]. Importantly, we observed expression in the AVM neuron, including during the L1 stage, when the AVM growth cone migrates ventrally. This expression pattern is consistent with our finding that *sdn-1*/syndecan expression in AVM (P*mec-7*::*sdn-1*) rescues the defects of *sdn-1* mutants (Fig 4C), supporting a cell-autonomous role for *sdn-1*/syndecan in AVM.
(TIF)

S5 Fig *lon-*2/glypican cannot be replaced by *sdn-1/*syndecan. *lon-2 slt-1* double mutants exhibit enhanced AVM guidance defects as compared to *slt-1* single mutants. The defects of the double mutants can be rescued back down to *slt-1* single mutant levels with expression of wild-type P*lon-2*::*lon-2*(+). In contrast, expression of *sdn-1(+)* in which *lon-2*/glypican is normally expressed (using the P*lon-2* promoter) or in the AVM neuron (using the heterologous promoter P*mec-7*) cannot rescue the axon guidance defects of *lon-2 slt-1* double mutants. Data for wild type, *lon-2*, *slt-1*, *lon-2 slt-1*, and P*lon-2*::*lon-2* in *lon-2 slt-1* are as in Fig 1B and 1C and Fig 4A. *** *p* ≤ 0.001, * *p* ≤ 0.05. (*z*-tests, *p*-values were corrected by multiplying by the number of comparisons) (see S2 and S3 Tables).
(TIF)

S6 Fig Detection of LON-2 and LON-2ΔGAG in the supernatant and cell extracts of S2 cell cultures by western blot analysis. (A) Diagram of LON-2/glypican variants expressed in S2 cells, HA::LON-2::myc and HA::LON-2ΔGAG::myc, in which the three HS attachment sites were mutated from Serine to Alanine residues. The core protein of LON-2/glypican is red, and the heparan sulfate chains (HS) are yellow. (B) In the supernatant of cells expressing HA::LON-2::myc, high molecular weight species were detected with the anti-myc antibody, which likely corresponds to full-length HA::LON-2::myc with HS chains attached. In contrast, the species detected in the supernatant of HA::LON-2ΔGAG::myc-expressing cells are smaller and fainter, indicating that HA::LON-2ΔGAG::myc indeed affects the synthesis of HS chains onto the LON-2/glypican core protein. No signal was detected with the anti-HA antibody in the supernatants, likely due to technical limitations. In the cell extracts from HA::LON-2::myc-expressing cells, the main species runs at ~90 kDa, and it is detected with both the anti-myc and anti-HA antibodies, suggesting that it is full length. This signal likely corresponds to the LON-2/glypican core protein devoid of HS chains, as it runs as a tight band. The slight mobility shift in cell extracts of HA::LON-2ΔGAG::myc-expressing cells compared to HA::LON-2::myc might correspond to a difference of mass and isoelectric point between HA::LON-2::myc and HA::LON-2ΔGAG::myc. Anti-actin and anti-HSP90 antibodies were used as loading controls. Representative blots of more than four independent repeats.
(TIF)

S7 Fig Detection of LON-2, UNC-40 and UNC-6 expression in S2 cells by western blot analysis. (A) Diagram of constructs used to express these proteins in S2 cells, showing the tags used to detect them. (B) Western blots for detection of HA::LON-2::myc, UNC-40::FLAG, and SfGFP::UNC-6. Constructs were individually and transiently transfected in S2 cells. Two d later, cells from single transfections were mixed and incubated overnight. Cells were harvested and combined with their corresponding supernatant from each of these cell mixes. Samples of each cell mix were split into three in order to run three parallel western blots and detect the proteins. As shown in S6 Fig, a main species (~90 kDa, bottom arrow) and high molecular weight species (top arrows) are detected with the anti-myc antibody against HA::LON-2::myc. As expected, UNC-40::FLAG and SfGFP::UNC-6 run at ~156 kDa and ~99 kDa, respectively. Representative blots of more than four independent repeats.
(TIF)

S8 Fig HA::LON-2 is released from cells (cotransfected with GFP) and associates with cells expressing UNC-40::FLAG. In order to distinguish HA::LON-2-producing cells from HA::LON-2-acquiring cells, two separate populations of cells were transfected. One population of S2 cells was transfected with UNC-40::FLAG. A second population of S2 cells was simultaneously transfected with both GFP and HA::LON-2. Two d later, the two populations of cells were mixed, incubated overnight, and immunostained with anti-HA and anti-FLAG antibodies, as described for Fig 6. In GFP-expressing cells (indicated by white asterisks), which had also been transfected with HA::LON-2, the HA::LON-2 signal was observed filling the cytoplasm. HA::LON-2 was also observed decorating the outline of UNC-40-expressing cells (indicated by white triangles, see Fig 6), supporting that LON-2/glypican associates with UNC-40-expressing cells. Occasionally, HA::LON-2 was observed on cells in which no UNC-40::FLAG was detected (indicated by the empty triangle). Scale bar 10 μm.
(TIF)

S9 Fig Controls for the specificity of the association of LON-2/glypican with UNC-40-expressing cells. (A) Experiments 1 and 2 show that HA::LON-2 does not associate with cells expressing the unrelated *Drosophila* type I transmembrane receptor Evi. Evi-expressing cells were mixed with cells expressing LON-2/glypican and/or UNC-40/DCC. As shown in experiment 2 and in Fig 6B and 6C, while LON-2/glypican associates with cells expressing UNC-40/DCC, LON-2/glypican does not associate with cells expressing Evi::GFP (experiments 1 and 2). Experiment 3 shows that SDN-1::myc/syndecan, another HSPG, does not associate with UNC-40/DCC-expressing cells. This SDN-1::myc was engineered to be secreted, as it lacks its transmembrane and intracellular C-terminal domains. These results indicate that the association of LON-2/glypican with UNC-40/DCC-expressing cells is specific and not a general feature of any HSPG. (B) Quantification of the association of HA::LON-2 with cells expressing UNC-40::FLAG, Evi::GFP, and untransfected cells. Ten different optical fields containing ~300 cells from three independent experiments were quantified and averaged. Error bars are standard error of the mean. Asterisks denote significant difference: *** *p* ≤ 0.001 (*t*-test versus untransfected cells). ns, not significant. (C) Quantification of the association of SDN-1::myc with cells expressing UNC-40::FLAG, HA::LON-2, and untransfected cells. Ten different optical fields containing ~300 cells from three independent experiments were quantified.
(TIF)

S1 Table List of mutant alleles used. (DOCX)

S2 Table AVM ventral guidance defects in strains examined, including the transgenic lines to rescue *sdn-1*. (DOCX)

S3 Table Transgenic rescue of *lon-2* function in AVM guidance. (DOCX)

S4 Table PVM dorsal guidance defects quantified in wild-type and mutant strains with or without misexpression of *unc-5* in the PVM neuron using transgene *evIs25* P*mec-7*::*unc-5*. (DOCX)

S5 Table Motorneuron axon dorsal guidance defects in strains examined. (DOCX)

S6 Table Distal tip cell guidance defects in strains examined. (DOCX)

S7 Table Transgenic rescue of *lon-2* function in distal tip cell guidance. (DOCX)

S8 Table List of strains used. (DOCX)

S9 Table List of primers used for building strains. (DOCX)

## Abbreviations

DTC: distal tip cell
FGF: fibroblast growth factor
GABA: gamma-aminobutyric acid
GFP: green fluorescent protein
GPI: glycerophosphatidylinositide
HA: human influenza hemagglutinin
HS: heparan sulfate
HSPG: heparan sulfate proteoglycan
NSA: netrin synergizing activity
RT-PCR: real-time PCR
SfGFP: superfolder GFP
TGFβ: transforming growth factor beta
