# Supplementary figures and images for: Glypican Is a Modulator of Netrin-Mediated Axon Guidance
Source: PLoS Biol. 2015 Jul 6;13(7):e1002183. doi: 10.1371/journal.pbio.1002183 (PMC4493048; doi:10.1371/journal.pbio.1002183)

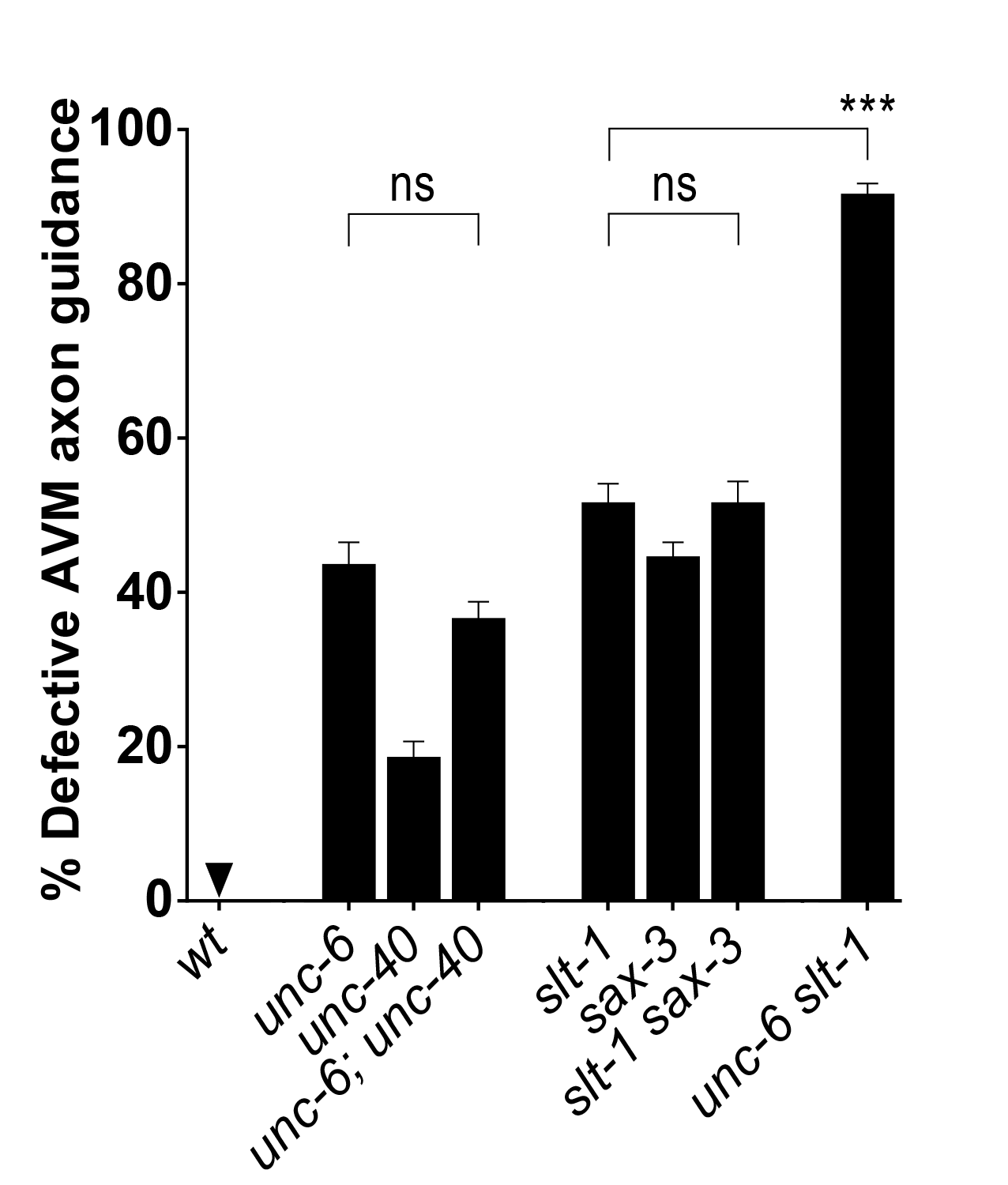

Supplement: S1 Fig — Mutations in unc-6/netrin and slt-1/slit pathways result in partially redundant defects, as previously established by the Culotti and Bargmann labs [13]. Mutants displayed here never exhibit a dorsally migrated AVM axon. Error bars are standard error of the proportion. Asterisks denote significant difference: *** p ≤ 0.001 (z-tests, p-values were corrected by multiplying by the number of comparisons). ns, not significant. (TIF) [file pbio.1002183.s003.tif]

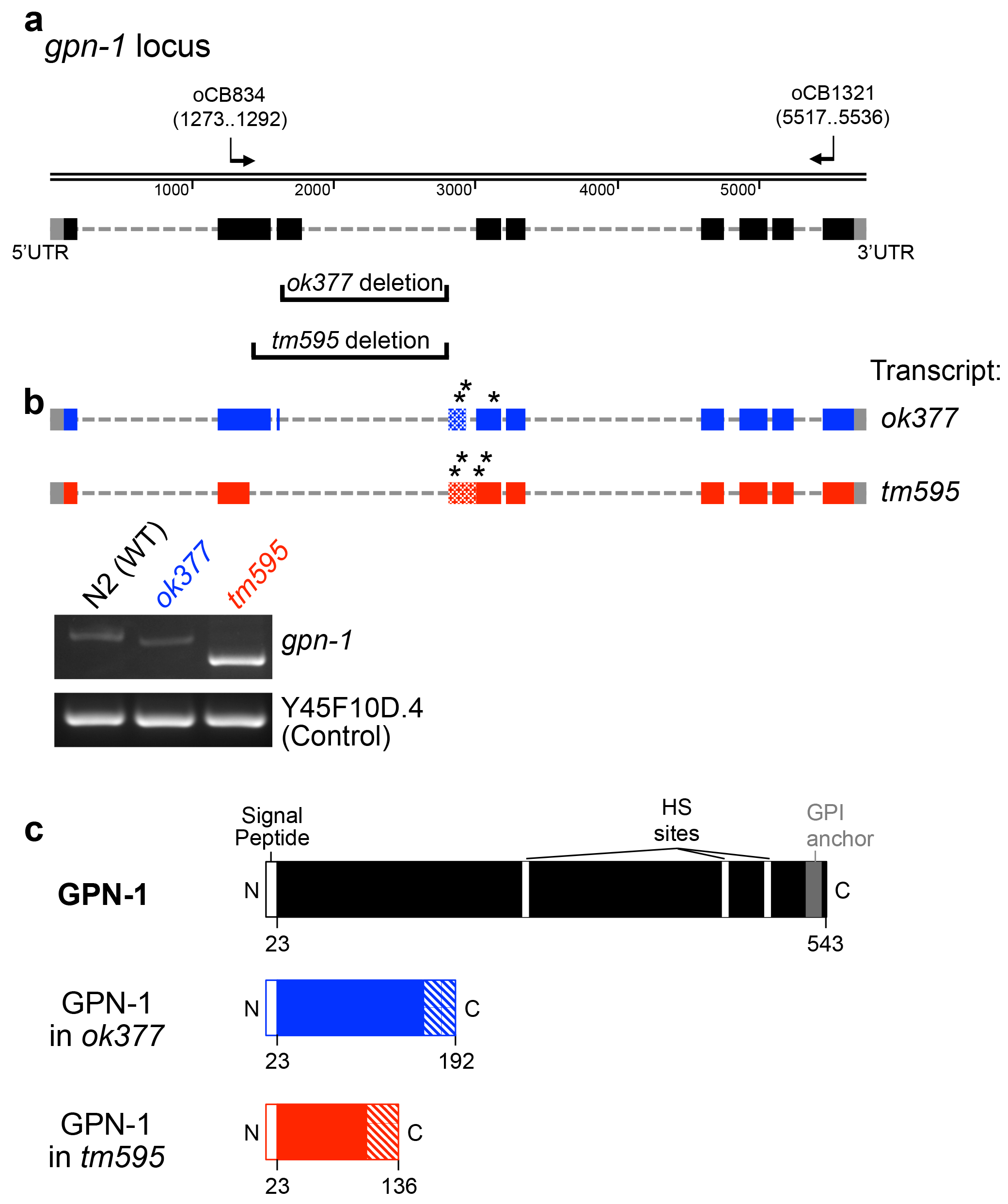

Supplement: S2 Fig — (A) gpn-1(ok377) and gpn-1(tm595) are deletions (brackets) in the gpn-1 locus. (B) RT-PCR using primers oCB834 and oCB1321 yields truncated products in gpn-1(ok377) and gpn-1(tm595). Y45F10D.4 is a housekeeping gene used as an RT-PCR control [55]. Sequencing of the gpn-1 RT-PCR products for ok377 (blue) reveals that the transcript lacks most of exon 3 and has several in-frame stop codons (*), as intronic sequence (hatch pattern) gets incorporated into the mature transcript. Sequencing of the gpn-1 RT-PCR product for tm595 (red) reveals that the transcript lacks exons 2 and 3 and has several in-frame stop codons (*), as intronic sequence (hatch pattern) gets incorporated into the mature transcript. No alternatively spliced products were detected in the mutants ok377 and tm595. (C) gpn-1(ok377) and gpn-1(tm595) are strong loss-of-function mutations, likely nulls, in which, at most, small truncated proteins would get produced. (TIF) [file pbio.1002183.s004.tif]

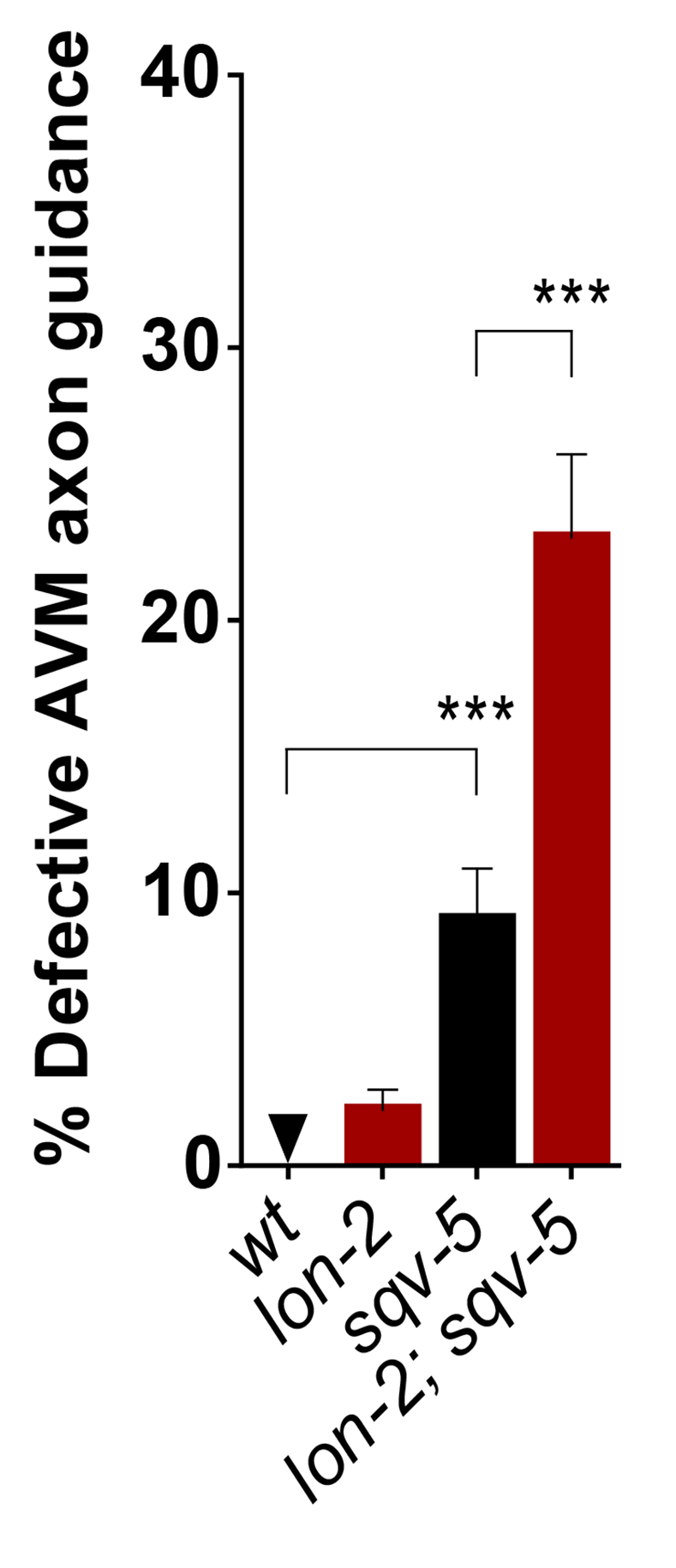

Supplement: S3 Fig — Loss of function of sqv-5, the gene coding for the chondroitin sulfate polymerase [61], leads to defective AVM ventral axon guidance, which is significantly enhanced by loss of lon-2 function. Error bars are standard error of the proportion. Asterisks denote significance: *** p ≤ 0.001 (z-tests, p-values were corrected by multiplying by the number of comparisons). (see also S2 Table). (TIF) [file pbio.1002183.s005.tif]

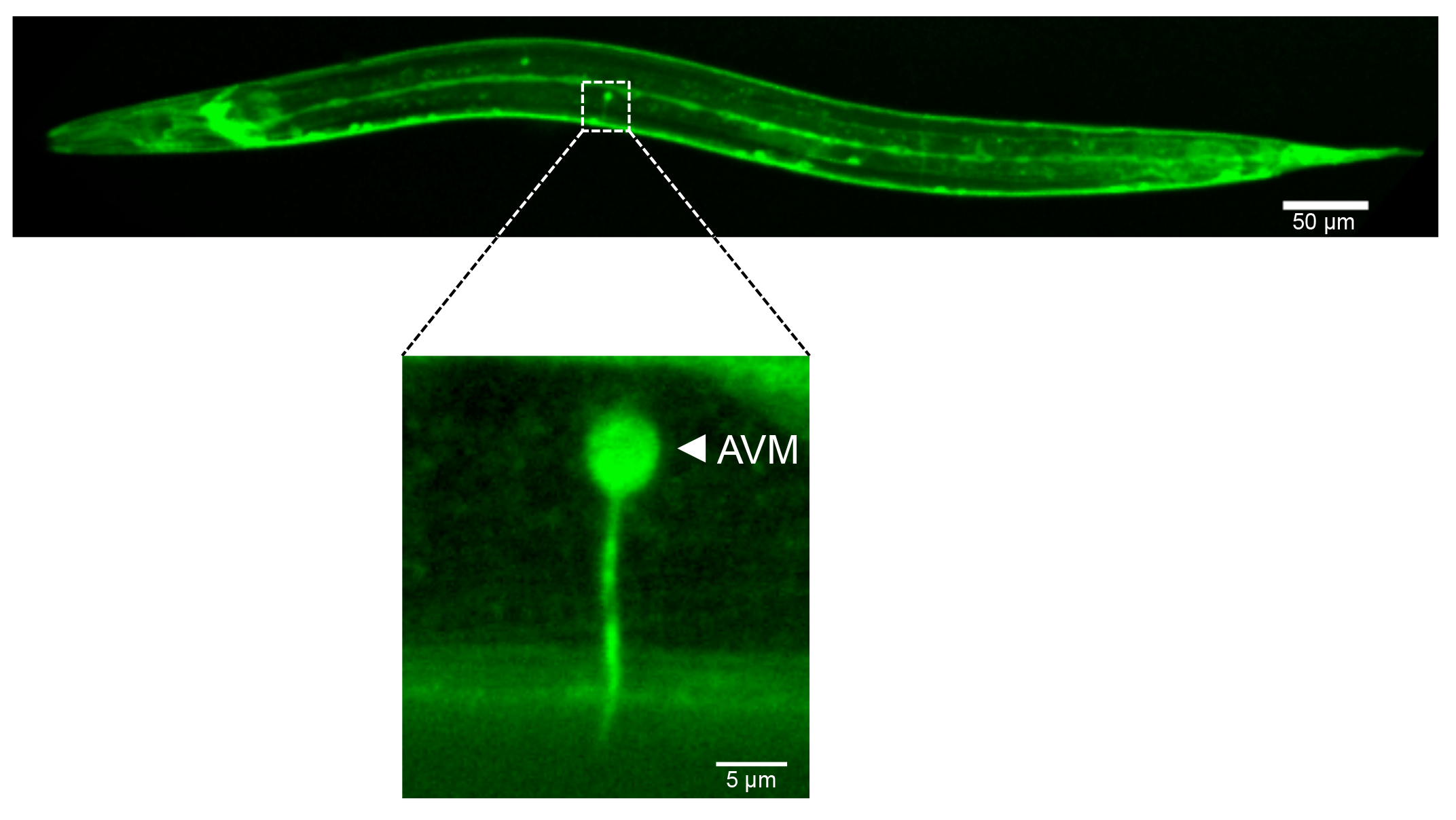

Supplement: S4 Fig — Using the translational fusion sdn-1::gfp (opIs171), we found that SDN-1::GFP is expressed in hypodermal cells and neurons, as previously reported [16]. Importantly, we observed expression in the AVM neuron, including during the L1 stage, when the AVM growth cone migrates ventrally. This expression pattern is consistent with our finding that sdn-1/syndecan expression in AVM (Pmec-7::sdn-1) rescues the defects of sdn-1 mutants (Fig 4C), supporting a cell-autonomous role for sdn-1/syndecan in AVM. (TIF) [file pbio.1002183.s006.tif]

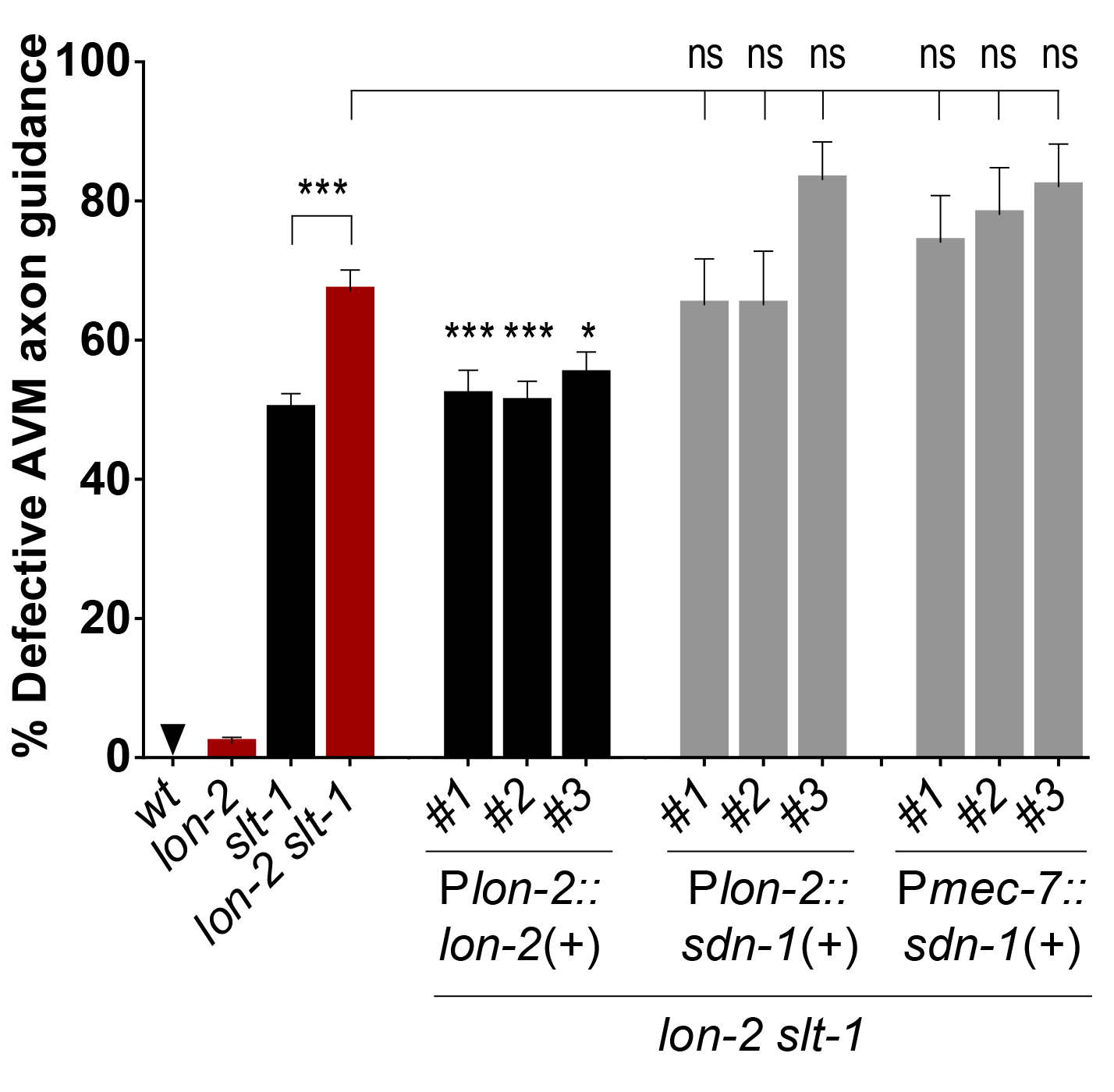

Supplement: S5 Fig — lon-2 slt-1 double mutants exhibit enhanced AVM guidance defects as compared to slt-1 single mutants. The defects of the double mutants can be rescued back down to slt-1 single mutant levels with expression of wild-type Plon-2::lon-2(+). In contrast, expression of sdn-1(+) in which lon-2/glypican is normally expressed (using the Plon-2 promoter) or in the AVM neuron (using the heterologous promoter Pmec-7) cannot rescue the axon guidance defects of lon-2 slt-1 double mutants. Data for wild type, lon-2, slt-1, lon-2 slt-1, and Plon-2::lon-2 in lon-2 slt-1 are as in Fig 1B and 1C and Fig 4A. *** p ≤ 0.001, * p ≤ 0.05. (z-tests, p-values were corrected by multiplying by the number of comparisons) (see S2 and S3 Tables). (TIF) [file pbio.1002183.s007.tif]

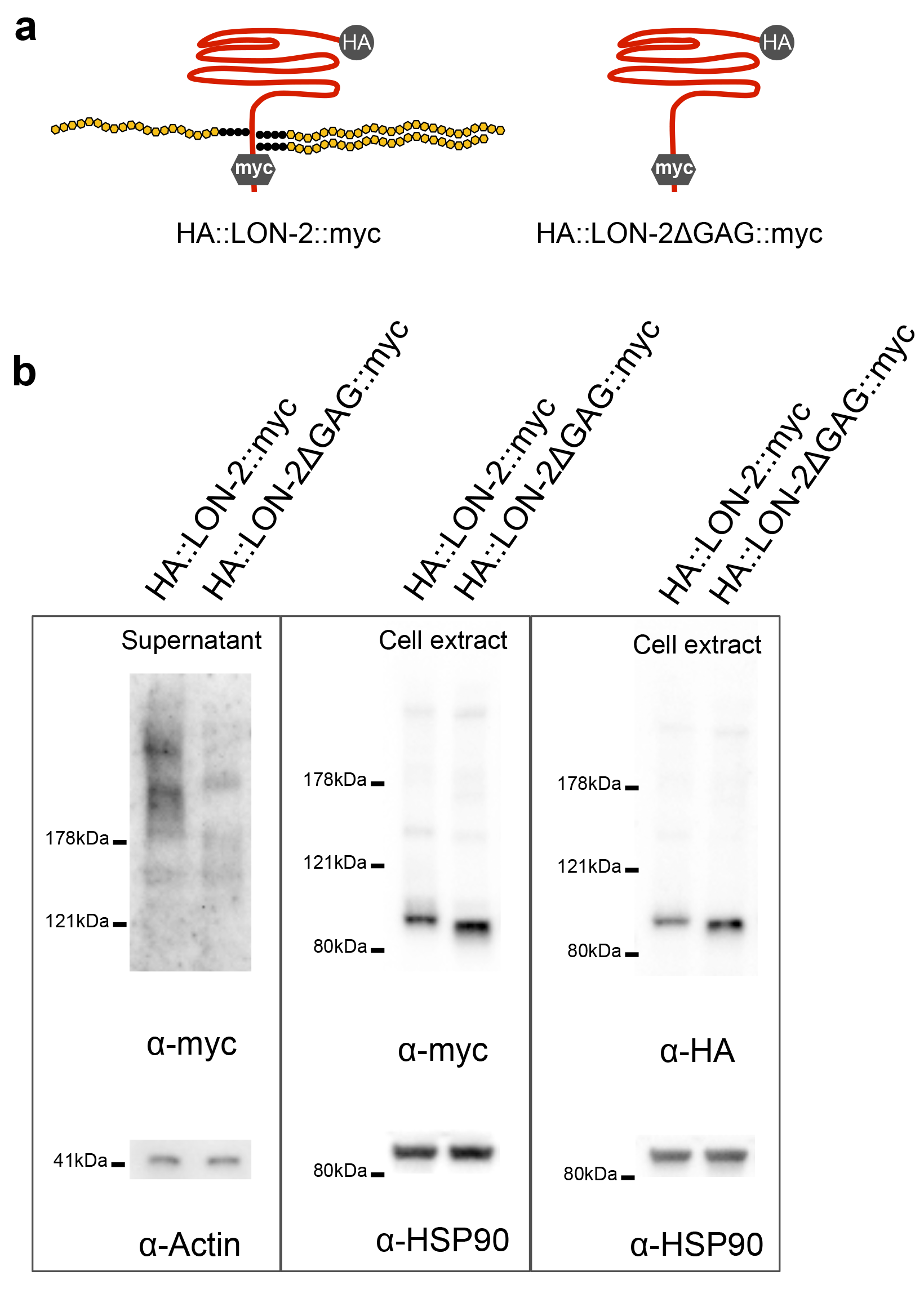

Supplement: S6 Fig — (A) Diagram of LON-2/glypican variants expressed in S2 cells, HA::LON-2::myc and HA::LON-2ΔGAG::myc, in which the three HS attachment sites were mutated from Serine to Alanine residues. The core protein of LON-2/glypican is red, and the heparan sulfate chains (HS) are yellow. (B) In the supernatant of cells expressing HA::LON-2::myc, high molecular weight species were detected with the anti-myc antibody, which likely corresponds to full-length HA::LON-2::myc with HS chains attached. In contrast, the species detected in the supernatant of HA::LON-2ΔGAG::myc-expressing cells are smaller and fainter, indicating that HA::LON-2ΔGAG::myc indeed affects the synthesis of HS chains onto the LON-2/glypican core protein. No signal was detected with the anti-HA antibody in the supernatants, likely due to technical limitations. In the cell extracts from HA::LON-2::myc-expressing cells, the main species runs at ~90 kDa, and it is detected with both the anti-myc and anti-HA antibodies, suggesting that it is full length. This signal likely corresponds to the LON-2/glypican core protein devoid of HS chains, as it runs as a tight band. The slight mobility shift in cell extracts of HA::LON-2ΔGAG::myc-expressing cells compared to HA::LON-2::myc might correspond to a difference of mass and isoelectric point between HA::LON-2::myc and HA::LON-2ΔGAG::myc. Anti-actin and anti-HSP90 antibodies were used as loading controls. Representative blots of more than four independent repeats. (TIF) [file pbio.1002183.s008.tif]

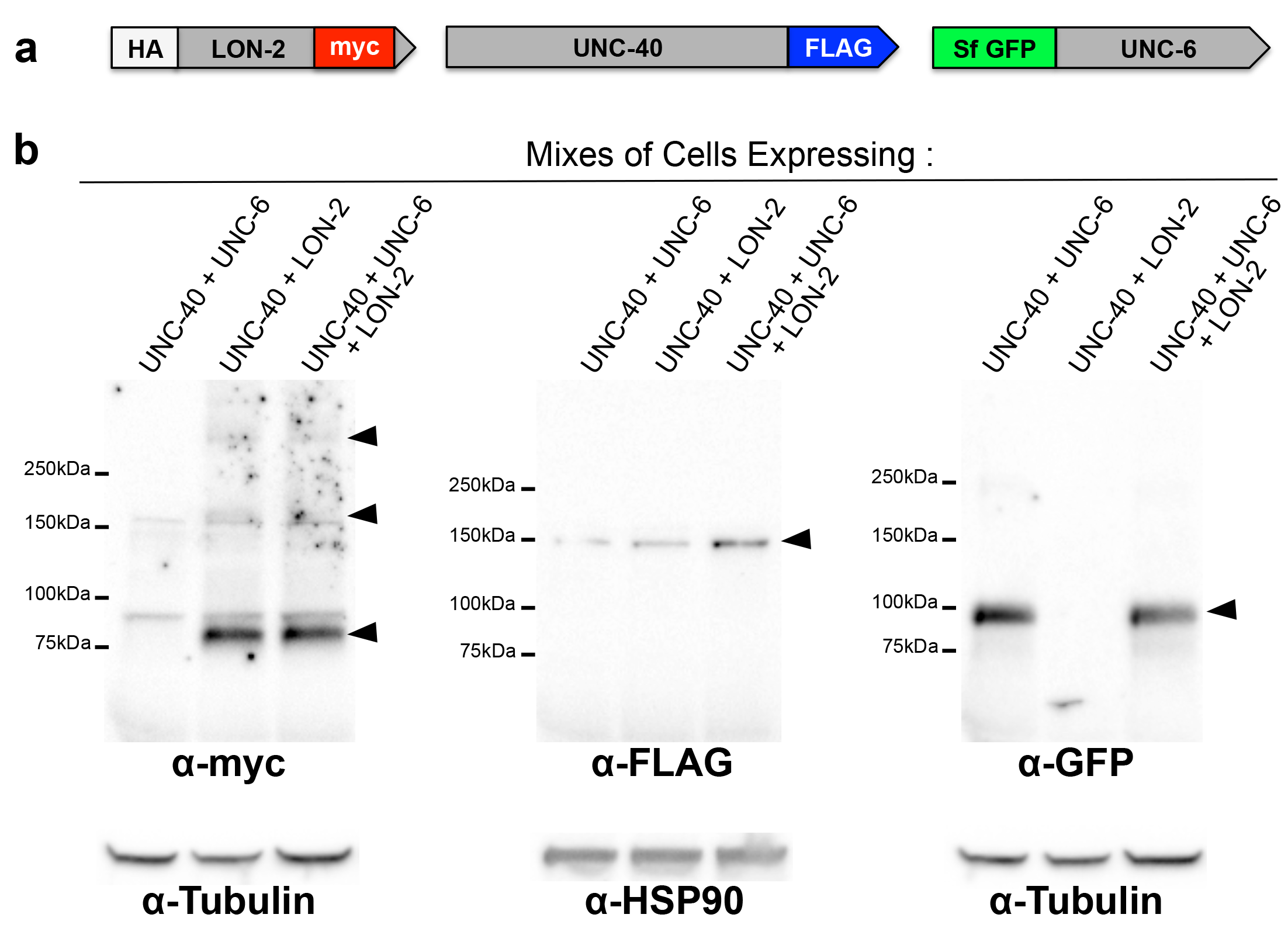

Supplement: S7 Fig — (A) Diagram of constructs used to express these proteins in S2 cells, showing the tags used to detect them. (B) Western blots for detection of HA::LON-2::myc, UNC-40::FLAG, and SfGFP::UNC-6. Constructs were individually and transiently transfected in S2 cells. Two d later, cells from single transfections were mixed and incubated overnight. Cells were harvested and combined with their corresponding supernatant from each of these cell mixes. Samples of each cell mix were split into three in order to run three parallel western blots and detect the proteins. As shown in S6 Fig, a main species (~90 kDa, bottom arrow) and high molecular weight species (top arrows) are detected with the anti-myc antibody against HA::LON-2::myc. As expected, UNC-40::FLAG and SfGFP::UNC-6 run at ~156 kDa and ~99 kDa, respectively. Representative blots of more than four independent repeats. (TIF) [file pbio.1002183.s009.tif]

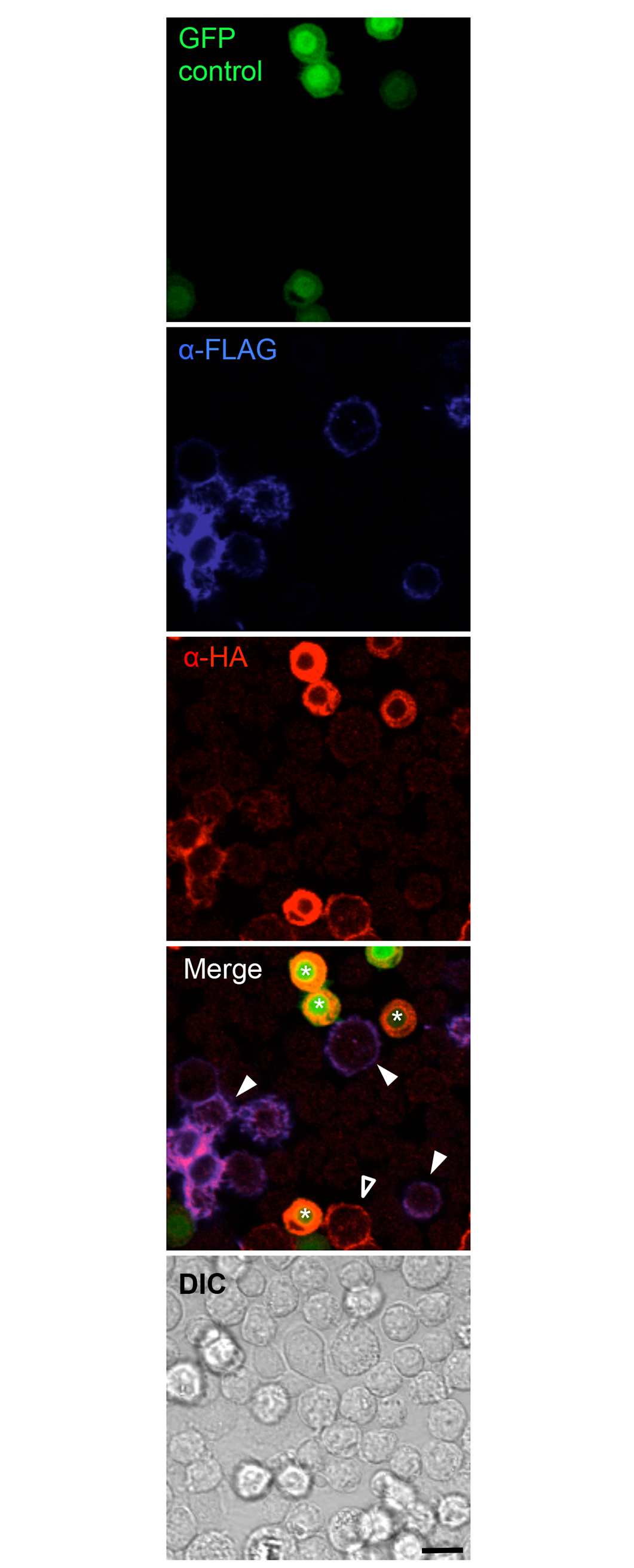

Supplement: S8 Fig — In order to distinguish HA::LON-2-producing cells from HA::LON-2-acquiring cells, two separate populations of cells were transfected. One population of S2 cells was transfected with UNC-40::FLAG. A second population of S2 cells was simultaneously transfected with both GFP and HA::LON-2. Two d later, the two populations of cells were mixed, incubated overnight, and immunostained with anti-HA and anti-FLAG antibodies, as described for Fig 6. In GFP-expressing cells (indicated by white asterisks), which had also been transfected with HA::LON-2, the HA::LON-2 signal was observed filling the cytoplasm. HA::LON-2 was also observed decorating the outline of UNC-40-expressing cells (indicated by white triangles, see Fig 6), supporting that LON-2/glypican associates with UNC-40-expressing cells. Occasionally, HA::LON-2 was observed on cells in which no UNC-40::FLAG was detected (indicated by the empty triangle). Scale bar 10 μm. (TIF) [file pbio.1002183.s010.tif]

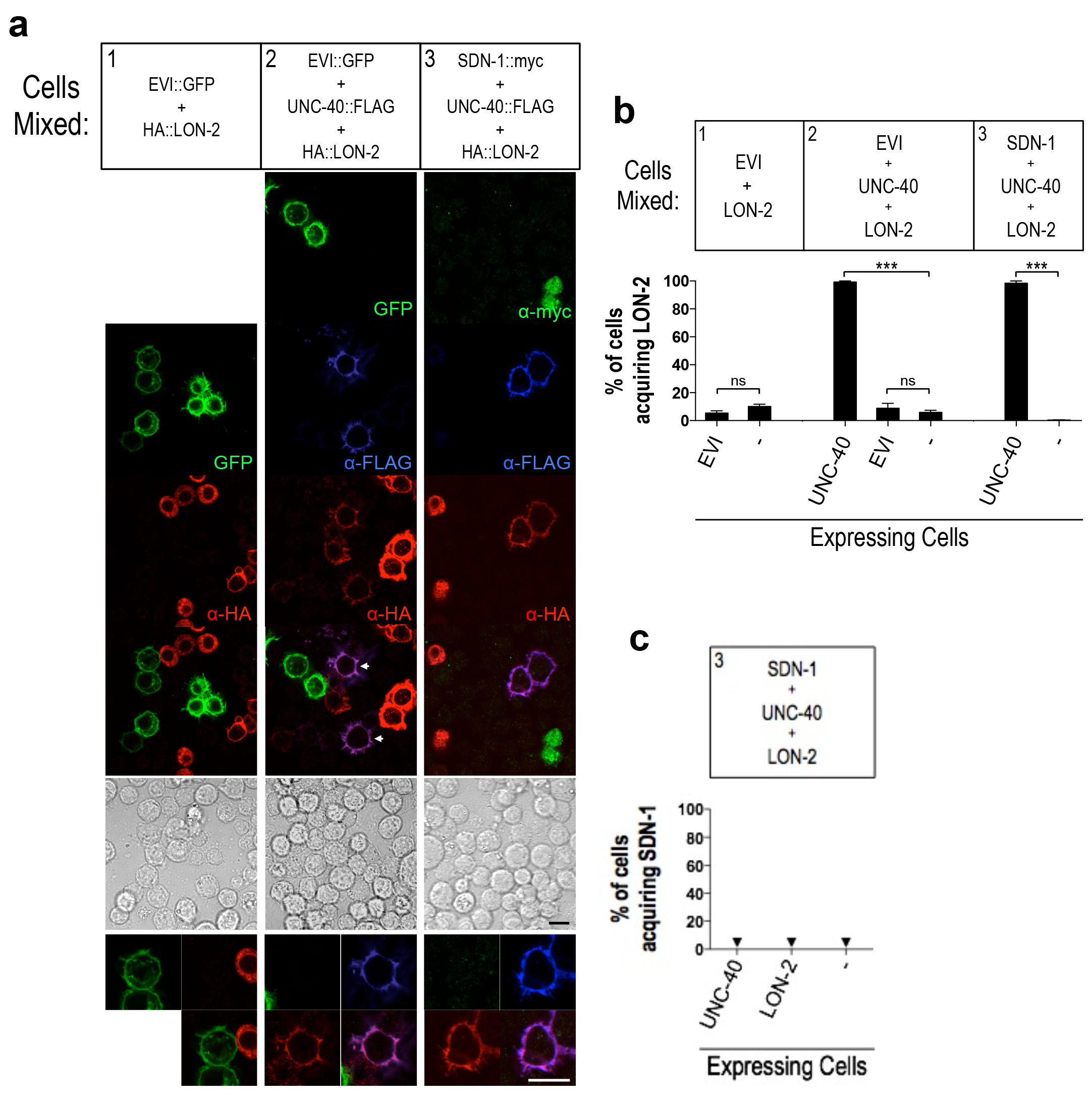

Supplement: S9 Fig — (A) Experiments 1 and 2 show that HA::LON-2 does not associate with cells expressing the unrelated Drosophila type I transmembrane receptor Evi. Evi-expressing cells were mixed with cells expressing LON-2/glypican and/or UNC-40/DCC. As shown in experiment 2 and in Fig 6B and 6C, while LON-2/glypican associates with cells expressing UNC-40/DCC, LON-2/glypican does not associate with cells expressing Evi::GFP (experiments 1 and 2). Experiment 3 shows that SDN-1::myc/syndecan, another HSPG, does not associate with UNC-40/DCC-expressing cells. This SDN-1::myc was engineered to be secreted, as it lacks its transmembrane and intracellular C-terminal domains. These results indicate that the association of LON-2/glypican with UNC-40/DCC-expressing cells is specific and not a general feature of any HSPG. (B) Quantification of the association of HA::LON-2 with cells expressing UNC-40::FLAG, Evi::GFP, and untransfected cells. Ten different optical fields containing ~300 cells from three independent experiments were quantified and averaged. Error bars are standard error of the mean. Asterisks denote significant difference: *** p ≤ 0.001 (t-test versus untransfected cells). ns, not significant. (C) Quantification of the association of SDN-1::myc with cells expressing UNC-40::FLAG, HA::LON-2, and untransfected cells. Ten different optical fields containing ~300 cells from three independent experiments were quantified. (TIF) [file pbio.1002183.s011.tif]
