# Supplementary material for: Glypican Is a Modulator of Netrin-Mediated Axon Guidance
Source: PLoS Biol. 2015 Jul 6;13(7):e1002183. doi: 10.1371/journal.pbio.1002183 (PMC4493048; doi:10.1371/journal.pbio.1002183)
Supplement: S1 Table — (DOCX) [file pbio.1002183.s012.docx]

| **Gene** | **Allele** | **Nature of allele** | **Reference** |
| --- | --- | --- | --- |
| *unc-52* | *e444* | Early stop in exon 18. Partial loss of function. | (Rogalski et al., 1995) (1) |
| *agr-1* | *tm2051* | 423 bp deletion, deleting exons 26 and 27 resulting in an in frame loss of 42 amino acids. | (Hrus et al., 2007) (2) |
| *gpn-1* | *ok377* | 1194 bp deletion, deletes most of exon 3, and introduces early Stop codons. Likely null. (see Figure S2) | (Hudson et al., 2006) (3)  This study |
| *gpn-1* | *tm595* | 1411 bp deletion, deletes part of exon 2, exon 3, and introduces early Stop codons. Likely null. (see Figure S2) | (Hudson et al., 2006) (3)  This study |
| *lon-2* | *e678* | ~9 kb deletion. Null. | (Gumienny et al., 2007) (4) |
| *sdn-1* | *zh20* | 1258 bp deletion. Null. | (Rhiner et al., 2005) (5) |
| *sdn-1* | *ok449* | 483 bp in-frame deletion. Produces truncated SDN-1. | (Minniti et al., 2004) (6) |
| *unc-6* | *ev400* | Early stop Q78*. Null. | (Wadsworth et al., 1996) (7) |
| *unc-6* | *e78* | C410Y. Partial loss of function. | (Lim and Wadsworth, 2002) (8) |
| *unc-40* | *e271* | Early stop R824*. Null. | (Stavoe et al., 2012) (9) |
| *unc-40* | *e1430* | Early stop R157*. Likely null. | (Colon-Ramos et al., 2007) (10) |
| *slt-1* | *eh15* | Duplication of locus and deletions. First copy contains a 1900 bp deletion. Both copies have a 100 bp deletion. First copy produces no mRNA while second copy produces mRNA with a frameshift. | (Hao et al., 2001) (11) |
| *sax-3* | *ky123* | Deletion of signal peptide and first exon. | (Zallen et al., 1998) (12) |
| *unc-34* | *e566* | A likely null mutation. | (Bloom, 1993) (13)  (Fleming et al., 2010) (14) |
| *sqv-5* | *k172* | G663E. Partial loss of function. | (Suzuki et al., 2006) (15) |

1. Rogalski TM, Gilchrist EJ, Mullen GP, Moerman DG. Mutations in the unc-52 gene responsible for body wall muscle defects in adult Caenorhabditis elegans are located in alternatively spliced exons. Genetics. 1995;139(1):159-69.

2. Hrus A, Lau G, Hutter H, Schenk S, Ferralli J, Brown-Luedi M, et al. C. elegans agrin is expressed in pharynx, IL1 neurons and distal tip cells and does not genetically interact with genes involved in synaptogenesis or muscle function. PLoS One. 2007;2(8):e731.

3. Hudson ML, Kinnunen T, Cinar HN, Chisholm AD. C. elegans Kallmann syndrome protein KAL-1 interacts with syndecan and glypican to regulate neuronal cell migrations. Dev Biol. 2006;294(2):352-65.

4. Gumienny TL, MacNeil LT, Wang H, de Bono M, Wrana JL, Padgett RW. Glypican LON-2 is a conserved negative regulator of BMP-like signaling in Caenorhabditis elegans. Curr Biol. 2007;17(2):159-64.

5. Rhiner C, Gysi S, Frohli E, Hengartner MO, Hajnal A. Syndecan regulates cell migration and axon guidance in C. elegans. Development. 2005;132(20):4621-33.

6. Minniti AN, Labarca M, Hurtado C, Brandan E. Caenorhabditis elegans syndecan (SDN-1) is required for normal egg laying and associates with the nervous system and the vulva. J Cell Sci. 2004;117(Pt 21):5179-90.

7. Wadsworth WG, Bhatt H, Hedgecock EM. Neuroglia and pioneer neurons express UNC-6 to provide global and local netrin cues for guiding migrations in C. elegans. Neuron. 1996;16(1):35-46.

8. Lim YS, Wadsworth WG. Identification of domains of netrin UNC-6 that mediate attractive and repulsive guidance and responses from cells and growth cones. J Neurosci. 2002;22(16):7080-7.

9. Stavoe AK, Nelson JC, Martinez-Velazquez LA, Klein M, Samuel AD, Colon-Ramos DA. Synaptic vesicle clustering requires a distinct MIG-10/Lamellipodin isoform and ABI-1 downstream from Netrin. Genes Dev. 2012;26(19):2206-21.

10. Colon-Ramos DA, Margeta MA, Shen K. Glia promote local synaptogenesis through UNC-6 (netrin) signaling in C. elegans. Science. 2007;318(5847):103-6.

11. Hao JC, Yu TW, Fujisawa K, Culotti JG, Gengyo-Ando K, Mitani S, et al. C. elegans Slit Acts in Midline, Dorsal-Ventral, and Anterior-Posterior Guidance via the SAX-3/Robo Receptor. Neuron. 2001;32(1):25-38.

12. Zallen JA, Yi BA, Bargmann CI. The conserved immunoglobulin superfamily member SAX-3/Robo directs multiple aspects of axon guidance in C. elegans. Cell. 1998;92(2):217-27.

13. Bloom L. Genetic and molecular analysis of genes required for axon outgrowth in Caenorhabditis elegans. 1993;Massachusetts Institute of Technology; Cambridge.

14. Fleming T, Chien SC, Vanderzalm PJ, Dell M, Gavin MK, Forrester WC, et al. The role of C. elegans Ena/VASP homolog UNC-34 in neuronal polarity and motility. Dev Biol. 2010;344(1):94-106.

15. Suzuki N, Toyoda H, Sano M, Nishiwaki K. Chondroitin acts in the guidance of gonadal distal tip cells in C. elegans. Dev Biol. 2006;300(2):635-46.
