# Supplementary material for: Glypican Is a Modulator of Netrin-Mediated Axon Guidance
Source: PLoS Biol. 2015 Jul 6;13(7):e1002183. doi: 10.1371/journal.pbio.1002183 (PMC4493048; doi:10.1371/journal.pbio.1002183)
Supplement: S2 Table — (DOCX) [file pbio.1002183.s013.docx]

| **Genotype** | |  | **N** | **% Defective** | **s.e.p.** |
| --- | --- | --- | --- | --- | --- |
| *zdIs5* | |  | 391 | **0** | 0.0 |
| **HSPG mutants** | | | | | |
| *lon-2(e678); zdIs5* | |  | 238 | **2** | 0.9 |
| *sdn-1(zh20); zdIs5* | |  | 506 | **12** | 1.4 |
| *sdn-1(ok449); zdIs5* | |  | 205 | **13** | 2.3 |
| *gpn-1(ok377); zdIs5* | |  | 58 | **0** | 0.0 |
| *gpn-1(tm595); zdIs5* | |  | 129 | **0** | 0.0 |
| *unc-52(e444); zdIs5* | |  | 190 | **2** | 1.0 |
| *agr-1(tm2051); zdIs5* | |  | 107 | **1** | 1.0 |
| *lon-2(e678) gpn-1(ok377); zdIs5* | |  | 110 | **1** | 0.9 |
| *lon-2(e678) gpn-1(tm595); zdIs5* | |  | 181 | **2** | 1.0 |
| *sdn-1(zh20) gpn-1(ok377); zdIs5* | |  | 222 | **13** | 2.3 |
| *sdn-1(zh20) gpn-1(tm595); zdIs5* | |  | 197 | **16** | 2.6 |
| *lon-2(e678) sdn-1(zh20); zdIs5* | |  | 283 | **33** | 2.8 |
| *lon-2(e678) sdn-1(ok449); zdIs5* | |  | 264 | **25** | 2.7 |
| *unc-52(e444); sdn-1(zh20); zdIs5* | |  | 131 | **12** | 2.8 |
| *unc-52(e444) agr-1(tm2051); zdIs5* | |  | 97 | **1** | 1.0 |
| *lon-2(e678) gpn-1(ok377) sdn-1(zh20); zdIs5* | |  | 218 | **41** | 3.3 |
| *lon-2(e678) gpn-1(tm595) sdn-1(zh20); zdIs5* | |  | 271 | **29** | 2.8 |
| **Strains with *unc-6* and *unc-40*** | | | | | |
| *unc-6(ev400); zdIs5* | |  | 199 | **43** | 3.5 |
| *unc-6(e78); zdIs5* | |  | 199 | **32** | 3.3 |
| *unc-40(e1430)* *zdIs5* | |  | 237 | **15** | 2.3 |
| *unc-40(e271) zdIs5* | |  | 190 | **18** | 2.8 |
| *unc-40(e271) zdIs5; unc-6(ev400)* | |  | 290 | **36** | 2.8 |
| *lon-2(e678) unc-6(ev400); zdIs5* | |  | 205 | **45** | 3.5 |
| *lon-2(e678) unc-6(e78); zdIs5* | |  | 262 | **28** | 2.8 |
| *unc-40(e271) zdIs5; lon-2(e678)* | |  | 246 | **18** | 2.4 |
| *unc-40(e1430) zdIs5; lon-2(e678)* | |  | 205 | **18** | 2.7 |
| *unc-40(e271) zdIs5; sdn-1(zh20)* | |  | 94 | **84** | 3.8 |
| *unc-6(ev400) slt-1(eh15); zdIs5* | |  | 188 | **91** | 2.1 |
| **Strains with *slt-1* and *sax-3*** | | | | | |
| *slt-1(eh15); zdIs5* | |  | 465 | **50** | 2.3 |
| *sax-3(ky123); zdIs5* | |  | 380 | **44** | 2.5 |
| *sax-3(ky123) slt-1(eh15); zdIs5* | |  | 220 | **51** | 3.4 |
| *lon-2(e678) slt-1(eh15); zdIs5* | |  | 233 | **67** | 3.1 |
| *sax-3(ky123) lon-2(e678); zdIs5* | |  | 493 | **53** | 2.2 |
| *sdn-1(zh20) slt-1(eh15); zdIs5* | |  | 378 | **41** | 2.5 |
| *sax-3(ky123) sdn-1(zh20); zdIs5* | |  | 137 | **45** | 4.3 |
| *kyIs209; zdIs5* | |  | 835 | **19** | 1.4 |
| *lon-2(e678) kyIs209; zdIs5* | |  | 783 | **25** | 1.5 |
| **Strains with *sqv-5*** | |  |  |  |  |
| *sqv-5(k172)* | |  | 235 | **9** | 1.9 |
| *lon-2(e678); sqv-5(k172)* | |  | 189 | **23** | 3.1 |
| **Transgenic strains used to rescue *sdn-1* function in AVM guidance in *lon-2 sdn-1; zdIs5*^** | | | | | |
|  | **Transgene** | |  |  |  |
| *lon-2(e678) sdn-1(zh20); zdIs5; qvEx114* | P*mec-7::sdn-1(+)* line #1 | | 129 | **14** | 3.1 |
| *lon-2(e678) sdn-1(zh20); zdIs5; qvEx115* | P*mec-7::sdn-1(+)* line #2 | | 136 | **15** | 3.1 |
| *lon-2(e678) sdn-1(zh20); zdIs5; qvEx100* | P*mec-7::sdn-1(+)* line #3 | | 99 | **14** | 3.5 |

N, number of AVM axons examined. s.e.p., standard error of the proportion.

^ We assayed rescue of *sdn-1* function using the double mutants *lon-2 sdn-1* since it is easier to rescue defects that are 33% penetrant (as in the double *lon-2(e678) sdn-1(zh20*)) than to rescue defects that are 12% penetrant (as in the single mutant *sdn-1(zh20)*).
