# Supplementary material for: Glypican Is a Modulator of Netrin-Mediated Axon Guidance
Source: PLoS Biol. 2015 Jul 6;13(7):e1002183. doi: 10.1371/journal.pbio.1002183 (PMC4493048; doi:10.1371/journal.pbio.1002183)
Supplement: S3 Table — (DOCX) [file pbio.1002183.s014.docx]

| **Genotype** |  |  | **N** | **% Defective** | **s.e.p** | **N** | **% Defective** | | **s.e.p** |
| --- | --- | --- | --- | --- | --- | --- | --- | --- | --- |
| *lon-2 slt-1* |  |  |  |  |  | 233 | **67** | | 3.1 |
| *slt-1* |  |  | 465 | **50** | 2.3 |  |  | |  |
|  | **Transgene** |  | **Transgenic** | | | **Non-transgenic sibling controls** | | | |
|  |  |  | **N** | **% Defective** | **s.e.p** | **N** | | **% Defective** | **s.e.p** |
| *lon-2 slt-1; qvEx107* | P*lon-2::lon-2(+)* (14kb *lon-2* locus) | line #1 | 184 | **52** | 3.7 | 221 | | **71** | 3.1 |
| *lon-2 slt-1; qvEx110* | P*lon-2::lon-2(+)* (P*lon-2::lon-2* cDNA) | line #2 | 263 | **51** | 3.1 | 223 | | **68** | 3.1 |
| *lon-2 slt-1; qvEx108* | P*lon-2::lon-2(+)* (P*lon-2::lon-2* cDNA) | line #3 | 223 | **55** | 3.3 | 190 | | **65** | 3.5 |
| *lon-2 slt-1; qvEx113* | P*elt-3::lon-2(+)* | line #1 | 116 | **47** | 4.6 | 71 | | **75** | 5.1 |
| *lon-2 slt-1; qvEx116* | P*elt-3::lon-2(+)* | line #2 | 248 | **52** | 3.2 | 231 | | **68** | 3.1 |
| *lon-2 slt-1; qvEx112* | P*dpy-7::lon-2(+)* | line #1 | 260 | **52** | 3.1 | 381 | | **65** | 2.4 |
| *lon-2 slt-1; qvEx117* | P*dpy-7::lon-2(+)* | line #2 | 592 | **54** | 2.0 | 281 | | **65** | 2.8 |
| *lon-2 slt-1; qvEx118* | P*dpy-7::lon-2(+)* | line #3 | 450 | **54** | 2.3 | 269 | | **64** | 2.9 |
| *lon-2 slt-1; qvEx184* | P*grd-10::lon-2(+)* | line #1 | 83 | **63** | 5.3 |  | | n.d. |  |
| *lon-2 slt-1; qvEx185* | P*grd-10::lon-2(+)* | line #2 | 99 | **65** | 4.8 |  | | n.d. |  |
| *lon-2 slt-1; qvEx186* | P*grd-10::lon-2(+)* | line #3 | 80 | **70** | 5.1 |  | | n.d. |  |
| *lon-2 slt-1; qvEx187* | P*mec-7::lon-2(+)* | line #1 | 81 | **62** | 5.4 |  | | n.d. |  |
| *lon-2 slt-1; qvEx188* | P*mec-7::lon-2(+)* | line #2 | 76 | **63** | 5.5 |  | | n.d. |  |
| *lon-2 slt-1; qvEx189* | P*mec-7::lon-2(+)* | line #3 | 99 | **70** | 4.6 |  | | n.d. |  |
| *lon-2 slt-1; qvEx190* | P*elt-2::lon-2(+)* | line #1 | 55 | **62** | 6.5 |  | | n.d. |  |
| *lon-2 slt-1; qvEx191* | P*elt-2::lon-2(+)* | line #2 | 49 | **67** | 6.7 |  | | n.d. |  |
| *lon-2 slt-1; qvEx192* | P*elt-2::lon-2(+)* | line #3 | 65 | **68** | 5.8 |  | | n.d. |  |
| *lon-2 slt-1; qvEx193* | P*myo-3::lon-2(+)* | line #1 | 52 | **63** | 6.7 |  | | n.d. |  |
| *lon-2 slt-1; qvEx194* | P*myo-3::lon-2(+)* | line #2 | 44 | **70** | 6.9 |  | | n.d. |  |
| *lon-2 slt-1; qvEx195* | P*myo-3::lon-2(+)* | line #3 | 54 | **78** | 5.6 |  | | n.d. |  |
| *lon-2 slt-1; qvEx204* | P*lon-2::sdn-1(+)* | line #1 | 51 | **65** | 6.7 |  | | n.d. |  |
| *lon-2 slt-1; qvEx205* | P*lon-2::sdn-1(+)* | line #2 | 37 | **65** | 7.8 |  | | n.d. |  |
| *lon-2 slt-1; qvEx206* | P*lon-2::sdn-1(+)* | line #3 | 47 | **83** | 5.5 |  | | n.d. |  |
| *lon-2 slt-1; qvEx207* | P*mec-7::sdn-1(+)* | line #1 | 42 | **74** | 6.8 |  | | n.d. |  |
| *lon-2 slt-1; qvEx208* | P*mec-7::sdn-1(+)* | line #2 | 37 | **78** | 6.8 |  | | n.d. |  |
| *lon-2 slt-1; qvEx209* | P*mec-7::sdn-1(+)* | line #3 | 39 | **82** | 6.2 |  | | n.d. |  |
| *lon-2 slt-1; qvEx121* | P*lon-2::*LON-2ΔGAG | line #1 | 233 | **49** | 3.3 | 184 | | 70 | 3.4 |
| *lon-2 slt-1; qvEx122* | P*lon-2::*LON-2ΔGAG | line #2 | 204 | **51** | 3.5 | 193 | | 74 | 3.2 |
| *lon-2 slt-1; qvEx196* | P*lon-2::*LON-2ΔGAG | line #3 | 158 | **53** | 4 | 215 | | 71 | 3.1 |
| *lon-2 slt-1; qvEx111* | P*lon-2::*LON-2ΔGPI | line #1 | 275 | **52** | 3.0 | 130 | | **75** | 3.8 |
| *lon-2 slt-1; qvEx199* | P*lon-2::*N-LON-2 | line #1 | 111 | **43** | 4.7 | 83 | | **71** | 5.0 |
| *lon-2 slt-1; qvEx173* | P*lon-2::*N-LON-2 | line #2 | 147 | **48** | 4.1 | 67 | | **75** | 5.3 |
| *lon-2 slt-1; qvEx174* | P*lon-2::*N-LON-2 | line #3 | 116 | **58** | 4.8 | 145 | | **74** | 3.6 |
| *lon-2 slt-1; qvEx176* | P*lon-2::*C-LON-2 | line #1 | 42 | **67** | 7.3 |  | | n.d. |  |
| *lon-2 slt-1; qvEx177* | P*lon-2::*C-LON-2 | line #2 | 52 | **67** | 6.5 |  | | n.d. |  |
| *lon-2 slt-1; qvEx178* | P*lon-2::*C-LON-2 | line #3 | 39 | **69** | 7.4 |  | | n.d. |  |

N, number of AVM axons examined. s.e.p, standard error of the proportion. n.d., not determined.
