# Supplementary material for: Glypican Is a Modulator of Netrin-Mediated Axon Guidance
Source: PLoS Biol. 2015 Jul 6;13(7):e1002183. doi: 10.1371/journal.pbio.1002183 (PMC4493048; doi:10.1371/journal.pbio.1002183)
Supplement: S4 Table — (DOCX) [file pbio.1002183.s015.docx]

| **Genotype** | **N** | **% Dorsal PVM axon ^** | **s.e.p.** |
| --- | --- | --- | --- |
| **PVM controls** | | | |
| *zdIs5* | 52 | **0** | 0.0 |
| *unc-6(ev400); zdIs5* | 44 | **0** | 0.0 |
| *unc-40(e271) zdIs5* | 30 | **0** | 0.0 |
| *unc-34(e566); zdIs5* | 73 | **0** | 0.0 |
| *lon-2(e678) unc-6(ev400); zdIs5* | 65 | **0** | 0.0 |
| *slt-1(eh15); zdIs5* | 60 | **0** | 0.0 |
| *sax-3(ky123); zdIs5* | 41 | **0** | 0.0 |
| *lon-2(e678); zdIs5* | 54 | **0** | 0.0 |
| *sdn-1(zh20); zdIs5* | 58 | **0** | 0.0 |
| *lon-2(e678) sdn-1(zh20); zdIs5* | 44 | **0** | 0.0 |
| **Strains with *evIs25* P*mec-7::unc-5*** | | | |
| *evIs25; zdIs5* | 228 | **66** | 3.1 |
| *unc-6(ev400) evIs25; zdIs5* | 41 | **0** | 0.0 |
| *unc-40(e271)* *zdIs5; evIs25* | 191 | **18** | 2.8 |
| *unc-34(e566); evIs25; zdIs5* | 159 | **13** | 2.7 |
| *unc-6(ev400) lon-2(e678)* *evIs25*; *zdIs5* | 62 | **0** | 0.0 |
| *unc-40(e271) zdIs5; lon-2(e678) evIs25* | 249 | **25** | 2.7 |
| *slt-1(eh15)* *evIs25*; *zdIs5* | 212 | **63** | 3.3 |
| *unc-34(e566); lon-2(e678) evIs25; zdIs5* | 207 | **8** | 1.9 |
| *sax-3(ky123)* *evIs25*; *zdIs5* | 201 | **66** | 3.3 |
| *lon-2(e678)* *evIs25; zdIs5* | 330 | **48** | 2.8 |
| *sdn-1(zh20) evIs25; zdIs5* | 259 | **64** | 3.0 |
| *lon-2(e678) sdn-1(zh20) evIs25; zdIs5* | 343 | **42** | 2.7 |

N, number of AVM axons examined. s.e.p., standard error of the proportion.

^ PVM axons normally never extend dorsally, not even in the complete absence of the *slt-1*/slit and the *unc-6*/netrin guidance pathways in *unc-6 slt-1* double null mutants, where axons defective in guidance extend *anteriorly*. Dorsal axon extension is only observed with *unc-5*/UNC5 ectopic expression, which overpowers the endogenous signaling mechanism within PVM and thus forces its axon to extend dorsally. This highlights the power of this ectopic-*unc-5*-expression system to uncover molecules specifically involved in *unc-6/*netrin signaling through the *unc-5/*UNC5 receptor, independently of other endogenous signals.
