# Supplementary material for: Glypican Is a Modulator of Netrin-Mediated Axon Guidance
Source: PLoS Biol. 2015 Jul 6;13(7):e1002183. doi: 10.1371/journal.pbio.1002183 (PMC4493048; doi:10.1371/journal.pbio.1002183)
Supplement: S5 Table — (DOCX) [file pbio.1002183.s016.docx]

| **Genotype** | **N** | **% GABA commissures failing to reach dorsal cord** | **s.e.p.** |
| --- | --- | --- | --- |
| *ufIs34; vsIs48* | 104 | **0.1** | 0.3 |
| *lon-2(e678); ufIs34* | 86 | **0.2** | 0.5 |
| *unc-40(e271); ufIs34; vsIs48* | 73 | **55** | 5.8 |
| *lon-2(e678); unc-40(e271); ufIs34* | 67 | **50** | 6.1 |
