# Supplementary material for: Glypican Is a Modulator of Netrin-Mediated Axon Guidance
Source: PLoS Biol. 2015 Jul 6;13(7):e1002183. doi: 10.1371/journal.pbio.1002183 (PMC4493048; doi:10.1371/journal.pbio.1002183)
Supplement: S6 Table — (DOCX) [file pbio.1002183.s017.docx]

| **Genotype** | **N** | **% Defective distal tip cell guidance** | **s.e.p.** |
| --- | --- | --- | --- |
| *ufIs34; vsIs48* | 140 | **4** | 1.7 |
| *lon-2(e678); ufIs34* | 251 | **17** | 2.4 |
| *unc-6(ev400); zdIs5* | 172 | **62** | 3.7 |
| *lon-2(e678) unc-6(ev400); zdIs5* | 160 | **65** | 3.8 |
| *unc-40(e271); ufIs34; vsIs48* | 122 | **25** | 3.9 |
| *lon-2(e678); unc-40(e271); ufIs34* | 136 | **22** | 3.6 |
| *unc-5(e53); vsIs48; ufIs34* | 134 | **46** | 4.3 |
| *lon-2(e678); unc-5(e53); ufIs34* | 138 | **49** | 4.3 |
