# Supplementary material for: Glypican Is a Modulator of Netrin-Mediated Axon Guidance
Source: PLoS Biol. 2015 Jul 6;13(7):e1002183. doi: 10.1371/journal.pbio.1002183 (PMC4493048; doi:10.1371/journal.pbio.1002183)
Supplement: S7 Table — (DOCX) [file pbio.1002183.s018.docx]

| **Genotype** | **Transgene** | **Transgenic** | | | **Non-transgenic sibling controls** | | |
| --- | --- | --- | --- | --- | --- | --- | --- |
|  |  | **N** | **% Defective** | **s.e.p** | **N** | **% Defective** | **s.e.p** |
| *lon-2(e678);ufIs34; qvEx200* | P*lon-2::lon-2(+)* (P*lon-2::lon-2* cDNA) | 182 | **5** | 1.6 | 100 | **18** | 3.8 |
| *lon-2(e678); texEx164* | P*lon-2::lon-2(+)* | 236 | **7** | 1.7 | 248 | **13** | 2.2 |
| *lon-2(e678); texEx144* | P*lon-2::*LON-2ΔGAG | 108 | **4** | 1.9 | 122 | **18** | 3.5 |
| *lon-2(e678);ufIs34; qvEx210* | P*lon-2::* N-LON-2 | 124 | **5** | 2.0 | 126 | **16** | 3.3 |
