# Supplementary material for: Glypican Is a Modulator of Netrin-Mediated Axon Guidance
Source: PLoS Biol. 2015 Jul 6;13(7):e1002183. doi: 10.1371/journal.pbio.1002183 (PMC4493048; doi:10.1371/journal.pbio.1002183)
Supplement: S8 Table — (DOCX) [file pbio.1002183.s019.docx]

| **Strain** | **Genotype** | | **Transgene** | **Reference** |
| --- | --- | --- | --- | --- |
| **Inserted transgenes** | | | | |
| N2 |  | |  | (Brenner, 1974)[15] |
| MT4005 | *zdIs5 I* | | P*mec-4::gfp; lin-15(+)* | (Clark and Chiu, 2003) [16] |
| VQ412 | *kyIs209 X* derived from CX5374 | | P*myo-3::slt-1* | (Yu et al., 2002) [17] |
| NW767 | *evIs25 X* | | P*mec-7::unc-5;* P*mec-7::lac-Z* | (Hamelin et al., 1993) [18] |
| WS3404 | *opIs171* | | P*sdn-1::sdn-1::gfp; lin-15(+)* | (Rhiner et al., 2005) [5] |
| LE311 | *lqIs4* | | P*ceh-10::gfp* | Tsalik 2003 |
| VQ84 | *vsIs48 X; ufIs34* derived from LX949 and IZ829 | | P*unc-17::gfp;* P*unc-47::mCherry* | (Chase et al., 2004)[19] (Petrash et al. 2013) [20] |
| **HSPG mutants** | | | | |
| VQ398 | *lon-2(e678) X; zdIs5 I* | |  | This study |
| VQ423 | *sdn-1(zh20) X; zdIs5 I* | |  | This study |
| VQ584 | *sdn-1(ok449) X; zdIs5 I* | |  | This study |
| VQ400 | *gpn-1(ok377) X; zdIs5 I* | |  | This study |
| VQ728 | *gpn-1(tm595) X; zdIs5 I* | |  | This study |
| VQ411 | *unc-52(e444) II; zdIs5 I* | |  | This study |
| VQ419 | *agr-1(tm2051) II; zdIs5 I* | |  | This study |
| VQ458 | *lon-2(e678) gpn-1(ok377) X; zdIs5 I* | |  | This study |
| VQ730 | *lon-2(e678) gpn-1(tm595) X; zdIs5 I* | |  | This study |
| VQ460 | *sdn-1(zh20) gpn-1(ok377) X; zdIs5 I* | |  | This study |
| VQ737 | *sdn-1(zh20) gpn-1(tm595) X; zdIs5 I* | |  | This study |
| VQ461 | *lon-2(e678) sdn-1(zh20) X; zdIs5 I* | |  | This study |
| VQ587 | *lon-2(e678) sdn-1(ok449) X; zdIs5 I* | |  | This study |
| VQ474 | *unc-52(e444) II; sdn-1(zh20) X; zdIs5 I* | |  | This study |
| VQ483 | *unc-52(e444) agr-1(tm2051) II; zdIs5 I* | |  | This study |
| VQ486 | *lon-2(e678) gpn-1(ok377) sdn-1(zh20) X; zdIs5 I* | |  | This study |
| VQ740 | *lon-2(e678) gpn-1(tm595) sdn-1(zh20) X; zdIs5 I* | |  | This study |
| VQ693 | *lon-2(e678) X; ufIs34* | |  | This study |
| **Strains with *unc-6*, *unc-40, unc-34, and unc-5*** | | | | |
| VQ396 | *unc-6(ev400) X; zdIs5 I* | |  | This study |
| VQ395 | *unc-6(e78) X; zdIs5 I* | |  | This study |
| VQ470 | *unc-40(e271)* *zdIs5 I* | |  | This study |
| VQ565 | *unc-40(e1430) zdIs5 I* | |  | This study |
| VQ529 | *unc-40(e271) zdIs5 I; unc-6(ev400) X* | |  | This study |
| VQ469 | *lon-2(e678) unc-6(ev400) X; zdIs5 I* | |  | This study |
| VQ567 | *lon-2(e678) unc-6(e78) X; zdIs5 I* | |  | This study |
| VQ522 | *unc-40(e271) zdIs5 I; lon-2(e678) X* | |  | This study |
| VQ581 | *unc-40(e1430) zdIs5 I; lon-2(e678) X* | |  | This study |
| VQ738 | *unc-40(e271) zdIs5 I; sdn-1(zh20) X* | |  | This study |
| VQ481 | *unc-6(ev400) slt-1(eh15) X; zdIs5 I* | |  | This study |
| VQ686 | *unc-40(e271) I; vsIs48 X; ufIs34* | |  | This study |
| VQ727 | *unc-40(e271) I; lon-2(e678) X; ufIs34* | |  | This study |
| VQ722 | *unc-34(e566) V; zdIs5 I* | |  | This study |
| VQ724 | *unc-5(e53) IV; vsIs48 X; ufIs34* | |  | This study |
| VQ723 | *unc-5(e53) IV; lon-2(e678) X; ufIs34* | |  | This study |
| **Strains with *slt-1* and *sax-3*** | | | | |
| VQ401 | *slt-1(eh15) X; zdIs5 I* | |  | This study |
| VQ473 | *sax-3(ky123) X; zdIs5 I* | |  | This study |
| VQ578 | *sax-3(ky123) slt-1(eh15) X; zdIs5 I* | |  | This study |
| VQ482 | *lon-2(e678) slt-1(eh15) X; zdIs5 I* | |  | This study |
| VQ501 | *sax-3(ky123) lon-2(e678) X; zdIs5 I* | |  | This study |
| VQ432 | *sdn-1(zh20) slt-1(eh15) X; zdIs5 I* | |  | This study |
| VQ526 | *sax-3(ky123) sdn-1(zh20) X; zdIs5 I* | |  | This study |
| VQ427 | *kyIs209 X; zdIs5 I* | |  | This study |
| VQ582 | *lon-2(e678) kyIs209 X; zdIs5 I* | |  | This study |
| **Strains with *evIs25*** | | | | |
| VQ536 | *evIs25 X; zdIs5 I* | |  | This study |
| VQ538 | *unc-6(ev400) evIs25 X; zdIs5 I* | |  | This study |
| VQ540 | *unc-40(e271)* *zdIs5 I; evIs25 X* | |  | This study |
| VQ555 | *unc-6(ev400) lon-2(e678)* *evIs25 X*; *zdIs5 I* | |  | This study |
| VQ645 | *unc-40(e271) zdIs5 I; lon-2(e678) evIs25 X* | |  | This study |
| VQ556 | *slt-1(eh15)* *evIs25 X*; *zdIs5 I* | |  | This study |
| VQ557 | *sax-3(ky123)* *evIs25 X*; *zdIs5 I* | |  | This study |
| VQ541 | *lon-2(e678)* *evIs25 X; zdIs5 I* | |  | This study |
| VQ572 | *sdn-1(zh20) evIs25 X; zdIs5 I* | |  | This study |
| VQ772 | *unc-34(e566) V; evIs25 X; zdIs5 I* | |  | This study |
| VQ773 | *unc-34(e566) V; evIs25 lon-2(e678) X; zdIs5 I* | |  | This study |
| VQ542 | *lon-2(e678) sdn-1(zh20) evIs25 X; zdIs5 I* | |  | This study |
| **Strains with *sqv-5*** | | | | |
| VQ523 | *sqv-5(k172) zdIs5 I* | |  | This study |
| VQ530 | *lon-2(e678) X; sqv-5(k172) zdIs5 I* | |  | This study |
| **Transgenic Lines** | | | | |
| VQ776 | *lon-2(e678) X; ufIs34; qvEx200* | pCB246 (P*lon-2::lon-2(+)*), P*ceh-22::gfp,* P*unc-122::rfp*. Line #1 | | This study |
| TLG257 | *lon-2(e678) X; texEx164* | Plasmid HW483 (P*lon-2::lon-2::gfp),* P*ttx-3::mCherry*. Line #2 | | This study |
| TLG199 | *lon-2(e678) X; texEx144* | pSBL3SG006 (P*lon-2::*LON-2ΔGAG::*gfp*), P*ttx-3::mCherry*. Line #1 | | This study |
| VQ795 | *lon-2(e678) X; qvEx210* | pCB270 (P*lon-2::*N-LON-2), pBSK+, P*ceh-22::gfp*, P*unc122::rfp*. Line #1 | | This study |
| VQ596 | *lon-2(e678) slt-1(eh15) X; zdIs5 I; qvEx107* | PCR product of bases 13,104 of cosmid C39E6 to 26,408 of F55D10, P*ceh-22::gfp,* P*unc-122::rfp.* Line #1 | | This study |
| VQ612 | *lon-2(e678) slt-1(eh15) X; zdIs5 I; qvEx110* | pCB246 (P*lon-2::lon-2*), P*ceh-22::gfp,* P*unc-122::rfp,* pBSK*+.* Line #2 | | This study |
| VQ597 | *lon-2(e678) slt-1(eh15) X; zdIs5 I; qvEx108* | pCB246 (P*lon-2::lon-2*), P*ceh-22::gfp,* P*unc-122::rfp,* pBSK*+.* Line #3 | | This study |
| VQ615 | *lon-2(e678) slt-1(eh15) X; zdIs5 I; qvEx113* | pCB218 (P*elt-3::lon-2*), P*ceh-22::gfp,* P*unc-122::rfp,* pBSK+. Line *#1* | | This study |
| VQ623 | *lon-2(e678) slt-1(eh15) X; zdIs5 I; qvEx116* | pCB218 (P*elt-3::lon-2*), P*ceh-22::gfp,* P*unc-122::rfp,* pBSK+. Line *#2* | | This study |
| VQ614 | *lon-2(e678) slt-1(eh15) X; zdIs5 I; qvEx112* | pCB268 (P*dpy-7::lon-2),* P*ceh-22::gfp,* P*unc-122::rfp;* pBSK+. Line #1 | | This study |
| VQ624 | *lon-2(e678) slt-1(eh15) X; zdIs5 I; qvEx117* | pCB268 (P*dpy-7::lon-2),* P*ceh-22::gfp,* P*unc-122::rfp,* pBSK+. Line #2 | | This study |
| VQ625 | *lon-2(e678) slt-1(eh15) X; zdIs5 I; qvEx118* | pCB268 (P*dpy-7::lon-2),* P*ceh-22::gfp,* P*unc-122::rfp,* pBSK+. Line #3 | | This study |
| VQ746 | *lon-2(e678) slt-1(eh15) X; zdIs5 I; qvEx184* | pCB266 (P*grd-10::lon-2)*, P*ceh-22:gfp,* P*unc-122::rfp,* pBSK+. Line #1 | | This study |
| VQ747 | *lon-2(e678) slt-1(eh15) X; zdIs5 I; qvEx185* | pCB266 (P*grd-10::lon-2)*, P*ceh-22:gfp,* P*unc-122::rfp,* pBSK+. Line #2 | | This study |
| VQ748 | *lon-2(e678) slt-1(eh15) X; zdIs5 I; qvEx186* | pCB266 (P*grd-10::lon-2)*, P*ceh-22:gfp,* P*unc-122::rfp,* pBSK+. Line #3 | | This study |
| VQ749 | *lon-2(e678) slt-1(eh15) X; zdIs5 I; qvEx187* | pCB251 (P*mec-7::lon-2)*, P*ceh-22:gfp,* P*unc-122::rfp,* pBSK+. Line #1 | | This study |
| VQ750 | *lon-2(e678) slt-1(eh15) X; zdIs5 I; qvEx188* | pCB251 (P*mec-7::lon-2)*, P*ceh-22:gfp,* P*unc-122::rfp,* pBSK+. Line #2 | | This study |
| VQ751 | *lon-2(e678) slt-1(eh15) X; zdIs5 I; qvEx189* | pCB251(P*mec-7::lon-2)*, P*ceh-22:gfp,* P*unc-122::rfp,* pBSK+. Line #3 | | This study |
| VQ752 | *lon-2(e678) slt-1(eh15) X; zdIs5 I; qvEx190* | pCB308 (P*elt-2::lon-2)*, P*ceh-22:gfp,* P*unc-122::rfp,* pBSK+. Line #1 | | This study |
| VQ753 | *lon-2(e678) slt-1(eh15) X; zdIs5 I; qvEx191* | pCB308 (P*elt-2::lon-2)*, P*ceh-22:gfp,* P*unc-122::rfp,* pBSK+. Line #2 | | This study |
| VQ754 | *lon-2(e678) slt-1(eh15) X; zdIs5 I; qvEx192* | pCB308 (P*elt-2::lon-2)*, P*ceh-22:gfp,* P*unc-122::rfp,* pBSK+. Line #3 | | This study |
| VQ755 | *lon-2(e678) slt-1(eh15) X; zdIs5 I; qvEx193* | pCB332 (P*myo-3::lon-2)*, P*ceh-22:gfp,* P*unc-122::rfp,* pBSK+. Line #1 | | This study |
| VQ756 | *lon-2(e678) slt-1(eh15) X; zdIs5 I; qvEx194* | pCB332 (P*myo-3::lon-2)*, P*ceh-22:gfp,* P*unc-122::rfp,* pBSK+. Line #2 | | This study |
| VQ757 | *lon-2(e678) slt-1(eh15) X; zdIs5 I; qvEx195* | pCB332 (P*myo-3::lon-2)*, P*ceh-22:gfp,* P*unc-122::rfp,* pBSK+. Line #3 | | This study |
| VQ621 | *lon-2(e678) sdn-1(zh20) X; zdIs5 I; qvEx114* | pCB242 (P*mec-7::sdn-1*), pRF4*,* P*ttx-3::mCherry.* Line #1 | | This study |
| VQ622 | *lon-2(e678) sdn-1(zh20) X; zdIs5 I; qvEx115* | pCB242 (P*mec-7::sdn-1*), pRF4*,* P*ttx-3::mCherry*. Line #2 | | This study |
| VQ507 | *lon-2(e678) sdn-1(zh20); zdIs5 I; qvEx100* | pCB242 (P*mec-7::sdn-1*), pRF4*,* P*ttx-3::mCherry.* Line #3 | | This study |
| VQ781 | *lon-2(e678) slt-1(eh15) X; zdIs5 I; qvEx204* | pCB312 (P*lon-2::sdn-1*), P*ceh-22::gfp*, P*unc-122::rfp*, pBSK+. Line #1 | | This study |
| VQ782 | *lon-2(e678) slt-1(eh15) X; zdIs5 I; qvEx205* | pCB312 (P*lon-2::sdn-1*), P*ceh-22::gfp*, P*unc-122::rfp*, pBSK+. Line #2 | | This study |
| VQ783 | *lon-2(e678) slt-1(eh15) X; zdIs5 I; qvEx206* | pCB312 (P*lon-2::sdn-1*), P*ceh-22::gfp*, P*unc-122::rfp*, pBSK+. Line #3 | | This study |
| VQ784 | *lon-2(e678) slt-1(eh15) X; zdIs5 I; qvEx207* | pCB242 (P*mec-7::sdn-1*), P*ceh-22::gfp*, P*unc-122::rfp*, pBSK+. Line #1 | | This study |
| VQ785 | *lon-2(e678) slt-1(eh15) X; zdIs5 I; qvEx208* | pCB242 (P*mec-7::sdn-1*), P*ceh-22::gfp*, P*unc-122::rfp*, pBSK+. Line #2 | | This study |
| VQ786 | *lon-2(e678) slt-1(eh15) X; zdIs5 I; qvEx209* | pCB242 (P*mec-7::sdn-1*), P*ceh-22::gfp*, P*unc-122::rfp*, pBSK+. Line #3 | | This study |
| VQ646 | *lon-2(e678) slt-1(eh15) X; zdIs5 I; qvEx121* | pSBL3SG006 (P*lon-2::*LON-2ΔGAG::*gfp*), P*ceh-22::gfp*, P*unc-122::rfp,* pBSK+. Line #1 | | This study |
| VQ647 | *lon-2(e678) slt-1(eh15) X; zdIs5 I; qvEx122* | pSBL3SG006 (P*lon-2::*LON-2ΔGAG::*gfp*), P*ceh-22::gfp*, P*unc-122::rfp,* pBSK+. Line #2 | | This study |
| VQ758 | *lon-2(e678) slt-1(eh15) X; zdIs5 I; qvEx196* | pSBL35G006 (P*lon-2::*LON-2ΔGAG::*gfp*), P*ceh-22::gfp*, P*unc-122::rfp,* pBSK+. Line #3 | | This study |
| VQ613 | *lon-2(e678) slt-1(eh15) X; zdIs5 I; qvEx111* | pCB269 (P*lon-2::* LON-*2*ΔGPI), P*ceh-22::gfp,* P*unc-122::rfp,* pBSK+*.* Line #1 | | This study |
| VQ761 | *lon-2(e678) slt-1(eh15) X; zdIs5 I; qvEx199* | pCB270 (P*lon-2::*N-LON-2), P*ceh-22::gfp*, P*unc-122::rfp,* pBSK+*.* Line #1 | | This study |
| VQ762 | *lon-2(e678) slt-1(eh15) X; zdIs5 I; qvEx173* | pCB270 (P*lon-2::*N-LON-2), P*ceh-22::gfp*, P*unc-122::rfp,* pBSK+*.* Line #2 | | This study |
| VQ763 | *lon-2(e678) slt-1(eh15) X; zdIs5 I; qvEx174* | pCB270 (P*lon-2::*N-LON-2), P*ceh-22::gfp*, P*unc-122::rfp,* pBSK+*.* Line #3 | | This study |
| VQ766 | *lon-2(e678) slt-1(eh15) X; zdIs5 I; qvEx176* | pCB311 (P*lon-2::*C-LON-2), P*ceh-22::gfp*, P*unc-122::rfp,* pBSK+*.* Line #1 | | This study |
| VQ767 | *lon-2(e678) slt-1(eh15) X; zdIs5 I; qvEx177* | pCB311 (P*lon-2::*C-LON-2), P*ceh-22::gfp*, P*unc-122::rfp,* pBSK+*.* Line #2 | | This study |
| VQ768 | *lon-2(e678) slt-1(eh15) X; zdIs5 I; qvEx178* | pCB311 (P*lon-2::*C-LON-2), P*ceh-22::gfp*, P*unc-122::rfp,* pBSK+*.* Line #3 | | This study |

Note: In this plasmid pSBL3SG006 (P*lon-2::lon-2(ΔGAG)*) all 3 GAG attachment sites of *lon-2* are mutated.
