# Supplementary material for: Glypican Is a Modulator of Netrin-Mediated Axon Guidance
Source: PLoS Biol. 2015 Jul 6;13(7):e1002183. doi: 10.1371/journal.pbio.1002183 (PMC4493048; doi:10.1371/journal.pbio.1002183)
Supplement: S9 Table — (DOCX) [file pbio.1002183.s020.docx]

| **Gene** | **Primer** | **Sequence** | **PCR product (bp)** | **Cosmid Coordinates** |
| --- | --- | --- | --- | --- |
| ***sdn-1(zh20)*** |  |  |  |  |
| Mutant specific | oCB837 | aaagagatgccggtcaggtg | 410 | F57C7 28510…28529, forward |
|  | oCB842 | aatggacgggatgagtgtcc |  | F57C7 26861…26880, reverse |
| Wild-type specific | oCB837 | aaagagatgccggtcaggtg | 293 | F57C7 28510…28529, forward |
|  | oCB876 | cttcagattcgagcctgctttgc |  | F57C7 28237…28259, reverse |
| ***sdn-1(ok449)*** |  |  |  |  |
| Mutant specific | oCB1114 | ttctgcctgtcgacttactc | 297 | F57C7 28453…28472, forward |
|  | oCB1125 | aagattgcggtaaaacacatc |  | F57C7 27692…27712, reverse |
| Wild-type specific | oCB1114 | ttctgcctgtcgacttactc | 478 | F57C7 28453…28472, forward |
|  | oCB1115 | ttcgtcgtcggttgggtagc |  | F57C7 27994…28013, reverse |
| ***gpn-1(ok377)*** |  |  |  |  |
| Mutant specific | oCB834 | atcaagaccgagtgatagtg | 501 | C03H12 6…25, forward |
|  | oCB843 | aatcatcagcatcgggaggg |  | C03H12 1682…1701, reverse |
| Wild-type specific | oCB834 | atcaagaccgagtgatagtg | 412 | C03H12 6…25, forward |
|  | oCB832 | ttttttgagggatatcatcg |  | C03H12 398…417, reverse |
| ***gpn-1(tm595)*** |  |  |  |  |
| Mutant specific | oCB1309 | agtcgattgcaaacgaatacg | 468 | F59D12 36353…36373 forward |
|  | oCB1310 | tcacacagtacgcttggcacg |  | C03H12 1822…1842 reverse |
| Wild-type specific | oCB1309 | agtcgattgcaaacgaatacg | 393 | F59D12 36353…36373 forward |
|  | oCB1311 | aagctttccatgcatactcgc |  | C03H12 356…376 reverse |
| ***agr-1(tm2051)*** |  |  |  |  |
| Mutant specific | oCB891 | cgaaaaatcgagagcaaaagg | 362 | F41G3 22649…22669, forward |
|  | oCB893 | tcagattcttgacacatccc |  | F41G3 21037…21056, reverse |
| Wild-type specific | oCB890 | tttgaactcttggacgaacc | 1210 | F41G3 22308…22327, reverse |
|  | oCB891 | cgaaaaatcgagagcaaaagg |  | F41G3 22649…22669, forward |
| ***slt-1(eh15)*** |  |  |  |  |
| Mutant specific | oCB919 | tatgacgtgttccggaaacc | 467 | C26G2 26145…26164, forward |
|  | oCB920 | atttctctaatacgggtagc |  | C26G2 28538…28557, reverse |
| Wild-type specific | oCB922 | tctcaattctaacatccatgtc | 339 | C26G2 28199…28220, forward |
|  | oCB920 | atttctctaatacgggtagc |  | C26G2 28538…28557, reverse |
| ***kyIs209:* Detection of insertion P*myo-3::slt-1*** | | |  |  |
|  | oCB945 | tcattcgggatattttgtgg | 592 |  |
|  | oCB950 | aagaagaagcatgcttctgg |  |  |
| ***sax-3(ky123)*** |  |  |  |  |
| Mutant specific | oCB1038 | agaatgtggctctctagtcc | ~330 | ZK377 15840…15859, forward |
|  | oCB1039 | tcgtttccgcgcattcagtc |  | ZK377 16580…16599, reverse |
| Wild-type specific | oCB1038 | agaatgtggctctctagtcc | 527 | ZK377 15840…15859, forward |
|  | oCB1042 | agcttcggattactgcttgc |  | ZK377 16348…16367, reverse |
| ***evIs25:* Detection of insertion P*mec-7::unc-5*** | | | | |
|  | oCB933 | ttgtcagtcgagcctcaagg | ~631 |  |
|  | oCB966 | tccactgtctgataatctgg |  |  |
| ***unc-40(e271)* sequencing** | | | | |
|  | oCB1077 | aattcgtgtaactgcttcc | 577 | T19B4 9418…9436, forward |
|  | oCB1076 | ttgaatatttcggaggttgc |  | T19B4 8859…8878, reverse |
| ***unc-40(e1430)* sequencing** | | | | |
|  | oCB1079 | atcaatgcgctgtacatgtg | 366 | T19B4 13106…13105, forward |
|  | oCB1078 | agagaccagggagttacagg |  | T19B4 12759…12778, reverse |
